# Extraordinary Diversity of Immune Response Proteins among Sea Urchins: Nickel-Isolated Sp185/333 Proteins Show Broad Variations in Size and Charge

**DOI:** 10.1371/journal.pone.0138892

**Published:** 2015-09-25

**Authors:** Lauren S. Sherman, Catherine S. Schrankel, Kristy J. Brown, L. Courtney Smith

**Affiliations:** 1 Department of Biological Sciences, George Washington University, Washington DC, United States of America; 2 Center for Genetic Medicine Research, Children’s National Medical Center, Washington, DC, United States of America; Instituto Nacional de Salud Pública, MEXICO

## Abstract

Effective protection against pathogens requires the host to produce a wide range of immune effector proteins. The *Sp185/333* gene family, which is expressed by the California purple sea urchin *Strongylocentrotus purpuratus* in response to bacterial infection, encodes a highly diverse repertoire of anti-pathogen proteins. A subset of these proteins can be isolated by affinity to metal ions based on multiple histidines, resulting in one to four bands of unique molecular weight on standard Western blots, which vary depending on the individual sea urchin. Two dimensional gel electrophoresis (2DE) of nickel-isolated protein samples followed by Western blot was employed to detect nickel-isolated Sp185/333 (Ni-Sp185/333) proteins and to evaluate protein diversity in animals before and after immune challenge with marine bacteria. Ni-Sp185/333 proteins of the same molecular weight on standard Western blots appear as a broad complex of variants that differ in pI on 2DE Western blots. The Ni-Sp185/333 protein repertoire is variable among animals, and shows a variety of changes among individual sea urchins in response to immune challenges with both the same and different species of bacteria. The extraordinary diversity of the Ni-Sp185/333 proteins may provide significant anti-pathogen capabilities for sea urchins that survive solely on innate immunity.

## Introduction

The purple sea urchin, *Strongylocentrotus purpuratus*, is a member of the echinoderm phylum this is a sister group to the chordates. It has an innate immune system that is complex and sophisticated [1] with a number of large gene families that mediate the immune function in these animals. One of these is the *Sp185/333* gene family with an estimate of up to 60 members that encode a highly diversified repertoire of putative immune response proteins [2–4]. These genes are upregulated following immune challenge with bacteria, lipopolysaccharide (LPS), peptidoglycan (PGN), β-1,3-glucan, and double stranded RNA [5–9]. The range of molecular weights (MWs) of the encoded Sp185/333 proteins is 4 to 55 kDa based on cDNA sequence predictions [7], however, most sea urchins express unexpectedly large Sp185/333 proteins that do not correspond to the predicted monomer MW [9,10]. When analyzed by two dimensional electrophoresis (2DE), up to 260 protein variants have been observed for an individual sea urchin with a range of MWs from 30 kDa to >200 kDa, and a range of isoelectric points (pI) of 3 to 10 [9], although this level of protein diversity is not observed for all animals [3]. Most of the Sp185/333^+^ spots on 2DE Western blots are acidic with a pI range of 3 to 6, and appear to vary in presence and intensity depending on the type of immune challenge [9]. Because these proteins are produced in response to pathogen contact and show significant sequence diversity, they may be an important aspect of the echinoid innate immune response. We have speculated that Sp185/333 proteins may have an expanded repertoire of immune responsiveness through six putative diversification mechanisms that include i) the presence of the large gene family both within individuals and within the population, which encodes a wide variety of protein isoforms, ii) putative variations in gene family size and gene sequence among individuals, iii) genomic instability within the *Sp185/333* gene family that may promote unequal crossovers, gene conversion, gene duplication/deletion and paralogous misspairing to promote sequence diversification, iv) variations in gene expression from different *Sp185/333* genes, v) RNA editing, perhaps by deaminases, or error prone transcription perhaps by low fidelity RNA polymerases, such as Polμ, that changes the amino acid sequence, alters the reading frame and inserts missense sequence, and changes single nucleotide changes that insert early stops leading to truncated proteins, and vi) a broad array of putative modifications to the proteins either during or after translation [2,3,9,11–13]. The outcome of these multiple layers of diversification is an array of the proteins that is much greater than expected from a gene family of up to 60 members. This wide range in proteins may enable swift and flexible activity against a wide range of pathogens, which is likely to be very effective in host protection in the marine system.

**Fig 1:**
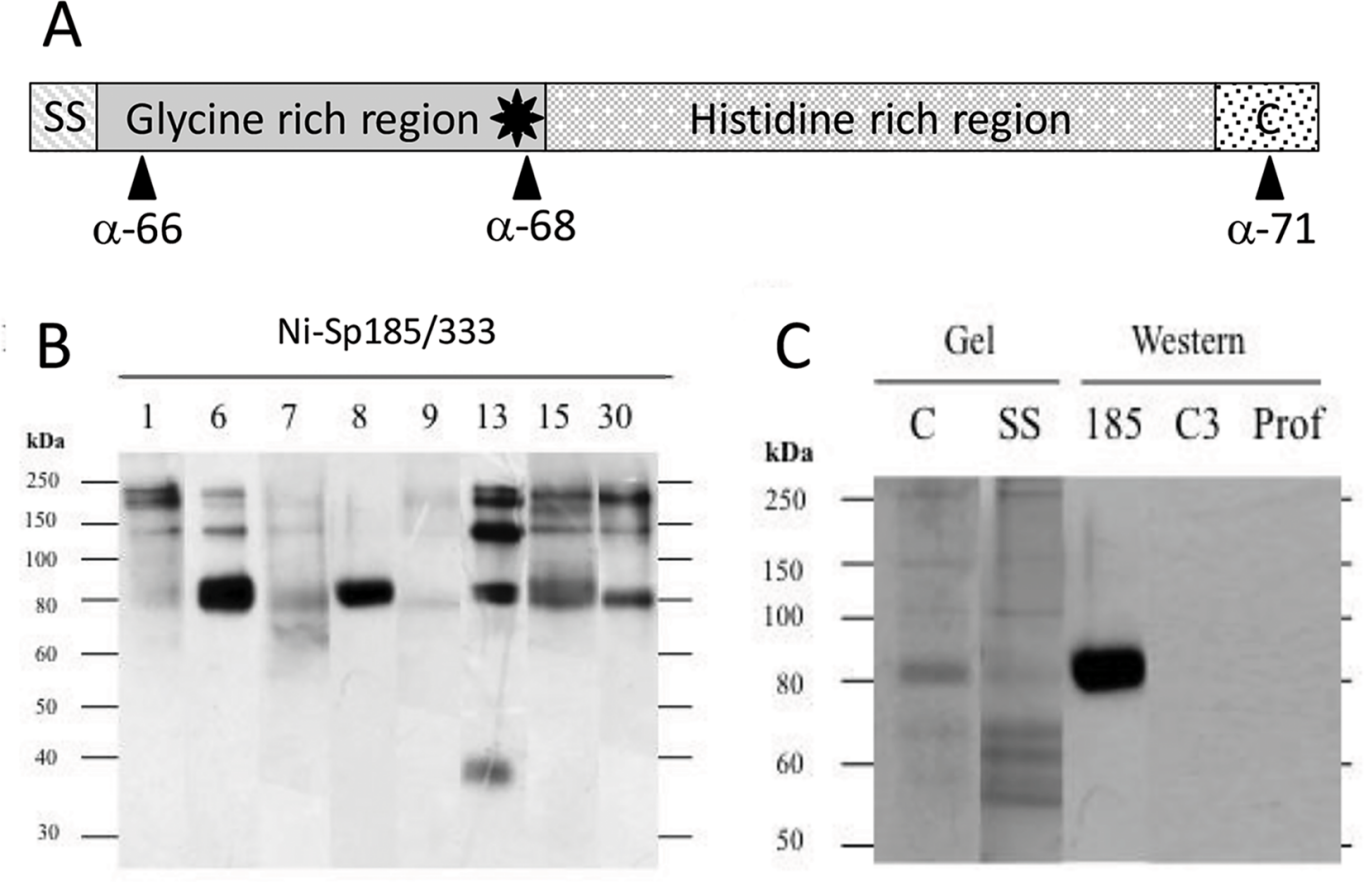
Sp185/333 protein structure and nickel affinity. (A) Sp185/333 protein structure. The Sp185/333 proteins have diverse sequences, however, their overall structure is conserved and includes a signal sequence (SS), which is likely cleaved during protein processing, a glycine-rich region including an arginine/glycine/aspartic acid (RGD) motif (star), a histidine-rich region, and a C terminal region (C). Three polyclonal rabbit anti-Sp185/333 sera against conserved peptides correspond to different regions of the protein (10). Anti-Sp185/333-66 (a-66) is specific for a region near the N terminus, anti-Sp185/333-68 (a-68) is specific for the region that includes the RGD motif, and anti-Sp185/333-71 (a-71) is specific for a moderately conserved region near the C terminus [9,10]. (B) Sp185/333 proteins can be isolated by affinity to Ni-His60 resin (ClonTech Laboratories, Inc.) based on the manufacturer’s instructions. A composite Western blot of elution fractions from eight sea urchins (designated by number at the top) are evaluated with a mixture of the three anti-Sp185/333 sera (1:15,000 dilution each), and several animals share proteins of the same size (see Table 2 for a list of all protein sizes in all samples). The 38 kDa band from animal 13 is likely the only monomer based on predictions from cDNA sequences [7]. (C) Sp185/333 proteins are eluted from nickel columns with low quantities of other coelomic fluid proteins. Elution fractions from animal 8 contain many protein bands when evaluated by Coomassie (C) and silver stain (SS). Analysis by Western blot shows that these elution fractions contain Sp185/333 proteins but are negative when probed for the sea urchin homologue of complement C3 with anti-SpC3 (C3, 1:8000 dilution [16]) or for the profilin homologue using anti-SpProfilin (Prof, 1:12000 dilution [17]).

Sp185/333 proteins are produced by the phagocyte class of coelomocytes (immune cells): small, polygonal and discoidal phagocytes in which the proteins are localized to the membranes of perinuclear vesicles, as well as to the extracellular surface of the plasma membrane of small phagocytes [10,14,15]. The level of Sp185/333 proteins in whole coelomic fluid (wCF; fluid plus cells) increases following immune challenge, as is evident from immunoquiescent sea urchins expressing little to no Sp185/333 proteins [10], while sea urchins challenged with LPS or PGN express many variants of Sp185/333 proteins in wCF [9]. The deduced Sp185/333 protein structure includes a signal sequence that is likely cleaved during protein processing, an N terminal glycine rich region including an arginine-glycine-aspartic acid (RGD) motif, a histidine rich region towards the C terminus, both tandem and interspersed imperfect repeats, histidine patches, regions of acidic amino acids, and no cysteines (Fig 1A). Although the Sp185/333 proteins are associated with membranes [10,14], the mechanism of association is not understood because the deduced, full-length proteins lack signatures for a transmembrane region or a conserved glycosylphosphatidylinositol linkage motif [8]. The amino acid sequences provide no clear folding predictions [8,10] and the proteins may be intrinsically disordered [Lun, Bishop, Smith; unpublished].

An analysis of the arrays of Sp185/333 proteins by 2DE/Western blots has been reported for a few sea urchins that indicated changes in the array for proteins of <64 kDa in response to challenge with LPS vs PGN [9]. Here, we expand on this initial report and evaluate changes in the Sp185/333 protein arrays from multiple sea urchins prior to immune challenge and after multiple challenges with several different isolates of marine bacteria. We have taken advantage of the predicted prevalence of histidines in the C terminal region of full-length Sp185/333 proteins to isolate a subset of native proteins from wCF by nickel affinity, which we have called Ni-Sp185/333 proteins. These are full-length proteins with sufficient histidines in the C terminal region to bind nickel. Other versions that are truncated, have missense sequence or insufficient histidines are not isolated by this approach. Evaluation of Ni-Sp185/333 proteins by standard Western blots identifies a set of bands with varying presence and intensity among individual sea urchins. However, it is not known whether these bands are composed of a single protein variant, or whether each band is a complex of proteins with the same MW but of varying pI. Using 2DE Western blots, we show that the Ni-Sp185/333 proteins of a few bands of different MW on 1DE Western blots are each composed of multiple protein variants with a range of pI. Furthermore, the repertoire of Ni-Sp185/333 proteins differs i) before vs. after immune challenge in individual sea urchins, ii) among individual sea urchins following immune challenge with the same bacteria, and iii) following multiple immune challenges among individual sea urchins. These results suggest that the Sp185/333 system in echinoids produces a highly diverse array of immune response proteins in the CF of sea urchins that may impart effective host protection, which is flexible for responding to the wide range of potential microbial pathogens that are present in the marine habitat.

## Materials and Methods

### Sea urchins

Adult purple sea urchins, *Strongylocentrotus purpuratus*, that were collected from the near shore waters of the Pacific ocean and purchased from Marinus Scientific Inc. (Long Beach, CA) or the Southern California Sea Urchin Co. (Corona del Mar, CA). Animals were maintained as described [16]. Individual animals were identified by placing them in individual, numbered plastic cages that were floated in the aquarium. A minimum of five animals were used throughout the time course experiments.

### Marine bacteria isolation and identification

One sea urchin was dissected and coelomocytes, gonad, peristomium, pharynx, and gut tissues were collected, swabbed and streaked onto Marine Broth (MB; 3.74% MB power, 0.3% yeast extract, 0.5% proteose peptone [Difco Laboratories]) plates and grown at room temperature (RT) for 2 days. Individual colonies of marine bacteria were re-streaked on separate plates, single colonies were grown in 2–5 ml MB cultures at RT with rotation, and genomic DNA was isolated according to [18]. As a positive control for sequencing, *Escherichia coli* (LE392 and XL1-Blue), was grown overnight in Luria Bertani (LB) broth at 37°C with rotation. Bacteria were pelleted and the cells were resuspended in 600 μl Tris-NaCl-EDTA-SDS-Urea buffer (TNESU; 10 mM Tris pH 7.4, 125 mM NaCl, 10 mM EDTA, 1% (w/v) SDS, 8 M Urea) plus proteinase K (6 μg/μl) and incubated at 37^°^C for 1–2 hr. The solution was extracted with phenol/sevag (50% phenol; 48% chloroform, 2% isoamyl alcohol), followed by sevag alone, precipitated with 2.5 M NH_4_Ac and 1 volume of isopropanol at -70°C, and centrifuged at 17,530 x *g* for 15 min at 4°C. DNA pellets were washed in ethanol, dried and resuspended in distilled water with RNAse A (ThermoFisher Scientific Inc.; 1–2 μg/50 μl). PCR amplification of the *16S* ribosomal RNA genes employed the 16Sfor (AGA GTT TGA TCC TGG CTC AG) and 16Srev (ACG GTT ACC TTG TTA CGA CTT) primers in a 50 μl reaction of 0.2 μM each primer, 200 μM deoxynucleotides, 1X company supplied buffer, 2.5 U ExTaq polymerase (Takara Bio Inc.), and 40 ng of bacterial genomic DNA. The PCR program was 95°C for 5 min followed by 25–30 cycles of 95°C for 1 min, 55°C for 1 min, 72°C for 1 min 30 sec, and a final extension at 72°C for 7 min. Amplicons were cleaned by the ExoSAP-IT kit (USB Corp.) to remove primers, sequenced with the 16Sfor primer using the BigDye Terminator cycle sequencing kit (Applied Biosystems) and evaluated on a CEQ8000 capillary sequencer (Beckman Coulter, Inc.). Multiple passes of the 16S sequence for each isolate were compared and assembled followed by searches of the non-redundant database at NCBI for matches to known bacterial sequences by BLASTn using Geneious R6 ver. 6.1.8 (Biomatters Ltd). Matches to bacterial *16S* sequences are listed in Table 1.

**Table 1:** Bacterial isolates^1^ from a dissected sea urchin that were available for immune challenges.

Isolate	Isolation tissue	Colony characteristics	BLASTn match to *16S* sequences from the nr database at NCBI^2^	BLAST evalue
C1.1		Yellow/tan, glossy	*Pseudoaltermonas sp*	0
C1.2	Coelomic fluid		*Pseudoaltermonas sp*	3.52e^-105^
C2.1		Off-white, glossy	*Vibrio tapetis*, *Vibrio sp*	0
Go1.1	Gonad	Off-white, glossy	*Vibrio sp*	0
Go1.2			*Vibrio sp*	0
Go2.1	Gonad	Light tan, glossy	*Vibrio sp*	0
Go2.2			*Vibrio sp*	0
Go3.1	Gonad	Off white, glossy	*Vibrio sp*	0
Go3.2		White, rough	*Vibrio sp*	0
G1.1	Gut	Off-white, glossy	*Vibrio sp*	0
G1.2			*Vibrio sp*	0
G2.1			*Pseudoaltermonas sp*	0
G2.2	Gut	Off white/tan, glossy	*Vibrio tapetis*, *Vibrio sp*	0
G2.3			*Pseudoaltermonas sp*	0
P1.1	Pharynx	Off white, glossy	*Idiomarina sp*	0
P1.2			*Idiomarina sp*	0
P2.1	Pharynx	pink, slightly glossy	*Shewanella sp*	0
P2.2			*Shewanella olleyana*, *Shewanella sp*	0
P3.1	Pharynx	White, rough	*Bacillus cereus*, *B*. *thuringiensis*	0

^1^Only colonies that grew on Marine Broth plates at room temperature were evaluated.

^2^Searches were done with Geneious R6 ver. 6.1.8.

### Bacteria selection for immune challenge

Initial immune challenges were conducted with *Vibrio diazotrophicus*, a marine bacterial species originally isolated from the green sea urchin, *Strongylocentrotus droebachiensis* (19) and obtained from the American Type Culture Collection (#33466), or were conducted with *E*. *coli*. Other species used for immune activation included Gram negative (*Vibrio tapetis*, isolate C2.1) and Gram positive bacteria (*Bacillus sp*, isolate P3.1) (Table 1). Overnight cultures of marine bacteria were heat-killed for 30 min at 95°C. To ensure the effectiveness of heat killing, bacteria were plated on MB plates and incubated for 48 hrs at RT to ensure that no colonies appeared and that *V*. *diazotrophicus*, which is flagellated and motile [19], showed no visible movement by microscopy over 2 min.

### Immune challenge and protein sample collection

Sea urchins were immunologically challenged either by injection of LPS (Sigma-Aldrich Co.; 1–2 μg/μl of artificial coelomic fluid [aCF: 10 mM CaCl_2_, 14 mM KCl, 50 mM MgCl_2_, 398 mM NaCl, 1.7 mM Na_2_HCO_3_, 25 mM Na_2_SO_4_ [7]) or with 10^4^ bacteria resuspended in 150 μl aCF. Volumes of LPS (1–2 μg/ml wCF) or bacteria (10^4^ cells/ml wCF) to inject were estimated by sea urchin wCF volume based on weight as described [20]. Animals were injected a second and third time with 10^6^ bacteria per ml of wCF at 24 hr and 48 hr after the initial challenge, or they received up to three injections of LPS (1–2 μg/ml wCF) at 24 hr intervals. wCF was withdrawn from the animals 24 hr after the third injection of heat-killed bacteria or the last LPS injection by inserting a 26 gauge needle through the peristomium, and aspirating 1.5 ml of wCF into a syringe preloaded with an ice chilled cocktail of protease inhibitors (10 mM benzamidine, 1 mM phenylmethanesulfonylfluoride [PMSF], 1x Protease Inhibitor Cocktail [Sigma-Aldrich]) in 50 mM Na_2_HPO_4_ pH 7.4 to block protein degradation.

### Protein isolation

Ni-Sp185/333 protein isolation by affinity to Ni-His60 resin for 1DE/Western blots initially followed the manufacturer’s instructions (ClonTech Laboratories, Inc.). The percentage of Ni-Sp185/333 proteins of total proteins in samples obtained by this protocol was analyzed by densitometry with a ChemiDoc XRS+ imaging system and associated software (Bio-Rad Laboratories, Inc.) by comparing Sp185/333^+^ bands on Western blots (see below) to identify the corresponding bands on Coomassie stained gels that were run in parallel.

Ni-Sp185/333 protein isolation was optimized by testing a variety of alternative approaches for cell lysis in addition to modifications to the nickel isolation protocol (S; S1 Table; S1 Fig). wCF diluted into the protease inhibitor cocktail (see above) was sonicated (amplitude 1–3%; Sonic Dismembrator 705; ThermoFisher), in the presence of detergent (1% CHAPS or 1% sarkosyl) to aid in cell lysis, increase protein solubility, prevent protein degradation, and prepare the samples for nickel isolation. Cell fragments were removed by centrifugation (7,000 x *g*, 30 min, 4°C) and the supernatant was passed through a nickel affinity column (bed volume 200 μl; Ni-His60, ClonTech). Binding and elution from nickel columns for Sp185/333 proteins was optimized by modifications to the imidazole concentration in the wash and elution buffers (S1 Protocol, S2 Fig). Unbound proteins were removed with 10 column volumes of wash buffer (10 mM NaCl, 50 mM Na_2_PO_4_, 10 mM imidazole), and the remaining bound proteins were eluted with 10 column volumes of elution buffer (10 mM NaCl, 50 mM Na_2_PO_4_, 300 mM imidazole). The wash and elution fractions were collected separately for each bed volume, and analyzed by 1DE Western blot (see below) to determine the fractions in which Ni-Sp185/333 proteins were present. These elution fractions were combined and used for further analysis. Traces of imidazole and salts were removed by buffer exchange and sample concentration (Amicron Ultra-4 Centrifugal Devices, 3 kDa MW cutoff; EMD Millipore Corp.) against distilled water. Samples were further purified by additional desalting by passage through a Micro Bio-Spin 6 chromatography column (Bio-Rad) into 10 mM Tris pH 7.4 based on the manufacturer’s instructions. Protein samples were dried by speed vacuum centrifugation in preparation for isoelectric focusing.

### Two dimensional gel electrophoresis (2DE)

The desalted, dried protein samples were dissolved in 1 ml of rehydration buffer (7 M urea, 2 M thiourea, 2% CHAPS, 0.5% Immobilized pH Gradient [IPG; GE Healthcare Life Sciences]) buffer and stored at -20°C. Methods were optimized by altering protein rehydration and introduction into the IEF strip, voltage employed for IEF, and the pH range of the strips (S1 Protocol, S3 Fig). Immediately before use, 50 mM dithiothreitol (DTT) was added to each sample and incubated at RT for 30 min to reduce thiols. Isoelectric focusing strips (pH 3–10 and pH 7–10, 11 cm, linear, Bio-Rad) were rehydrated with 200 μl of dissolved protein by active rehydration (50 V, 12 hr) in a Protean IEF Cell (Bio-Rad). Constant sample volume was used rather than constant protein concentration because the protein samples included echinochrome [21] that often co-eluted with the Sp185/333 proteins and prevented accurate measurement of protein concentration. The strips were focused to separate the nickel-isolated proteins by differences in pI as previously described [22] and as optimized (S1 Protocol). Following the first dimension, the IEF strips were held at 500 V until samples were separated by MW using 4–15% gradient TGX polyacrylamide gels (Bio-Rad) as described [22] with minor changes. Briefly, focused strips were equilibrated for 30 min in equilibration buffer (50 mM Trizma pre-set crystals, pH 8.8, 6 M urea, 30% glycerol, 2% SDS, 0.002% bromophenol blue) plus 1% DTT, followed by alkylation of all cysteines and thiols by incubation in equilibration buffer with 2% iodoacetamide. The equilibrated strip was placed in the top of the TGX polyacrylamide gel and covered with ReadyPrep Overlay Agarose (Bio-Rad). Once set, the gel was run at 200 V, constant voltage, for 45 min to 1 hr at 4°C. Duplicate 2DE gels analyzed by Western blots (see below) were used to demonstrate that processing and storage did not result in artifacts to alter spot positions (S1 Protocol; S3 Fig).

### Western blots

After the proteins were separated in the second dimension, the gel was electro-blotted to transfer the proteins onto a polyvinylidene fluoride (PVDF) membrane using a Trans-Blot SD Semi-Dry Transfer Cell (Bio-Rad) in transfer buffer (25 mM Tris Base pH 8.3, 192 mM glycine, 20% methanol). After transfer, the membrane was rinsed in Tris-NaCl (TN, 25 mM Tris pH 7.4, 0.5 M NaCl) followed by Tris-NaCl-Tween (TNT; TN with 0.1% Tween 20), and blocked with Blotto (5% [w/v] powdered milk in TNT) overnight at RT. Blotto was replaced with fresh Blotto containing a cocktail of three anti-Sp185/333 antibodies, which were specific to different conserved regions of the Sp185/333 proteins (Fig 1A) as previously described [9,10]. Each antibody was diluted 1:15,000 for 1DE Western blots or 1:7,500 for 2DE Western blots. After washing in TNT, membranes were incubated with goat anti-rabbit immunoglobulins conjugated with horseradish peroxidase (GaR-Ig-HRP, 1:30,000 in Blotto; ThermoFisher) for 1 hr. After washing twice in TNT and once in TN, filters were developed in either Western Lighting ECL reagent (PerkinElmer Inc.) for 1DE Western blots or SuperSignal West Pico Chemiluminescent Substrate (ThermoFisher) for 2DE Western blots followed by exposure to X-ray film or evaluated on a ChemiDoc XRS+ (Bio-Rad).

### Peptide preparation for mass spectrometry

Following 2DE, gels were stained with BioSafe Coomassie Stain (Bio-Rad). In preparation, gels were soaked in fixing solution (50% methanol, 5% acetic acid) for 30 min, stained with BioSafe Coomassie Stain for 1 hr, and destained in double distilled water at 4°C overnight. Gels were scanned and individual spots chosen for mass spectrometric analysis were cut from the gel and digested with trypsin as previously described [22]. Briefly, multiple cycles of dehydration in 50% acetonitrile followed by rehydration in 50 mM NH_4_HCO_3_ was done to remove the SDS and protein stains from the gel pieces. Following the last wash and dehydration cycle, gel pieces were rehydrated in 50 mM NH_4_HCO_3_ with 12.5 ng/μl trypsin (sequencing grade) and incubated for 45 min on ice followed by 37°C overnight. Digested peptides were extracted from the gel pieces with 25 mM NH_4_HCO_3_, acetonitrile, and 10% formic acid and dried by vacuum centrifugation.

### Protein identification: ESI-LTQ-MS/MS

Peptides recovered from digested 2DE gel fragments were analyzed using nanospray electrospray ionization (ESI) LTQ linear iontrap (LTQ) tandem mass spectrometry (MS/MS) (ThermoFisher) as previously described [23] with minor changes. Briefly, peptides were loaded onto a C18 reverse-phase column (3.5 μm, 75 μm x 15 cm; ThermoFisher) to concentrate and desalt the samples prior to being separated and eluted. The LTQ performed one full MS scan (300–2,000 m/z) to select the five most intense peaks through dynamic exclusion for MS/MS analysis via collision-induced dissociation with helium. Resulting MS raw files were searched against the NCBI protein database for *Strongylocentrotus purpuratus* containing 38,417 entries (downloaded May 2015) using Proteome Discoverer software (ThermoFisher) with the following parameters: semi-tryptic peptides, up to two missed cleavages, precursor mass tolerance of 1.5 Da, product ion mass tolerance of 1 Da, possible oxidation of methionine (15.995 Da). The resulting protein matches were filtered with the following parameters: at least two peptides per protein, ΔCN >0.1, Xcorr vs charge state of 1.5 for z = 1, Xcorr = 2 for z = 2, Xcorr = 2.25 for z = 3 and Xcorr = 2.5 for z = 4, and the Proteome Discoverer software Percolator [24,25] Target FDR <1%.

### Spot detection

Images of the 2DE Western blots were saved as TIFF files for analysis using Quantity One 1-D and spot identification with PDQuest analysis software (Bio-Rad). Protein density was quantified by dividing each membrane into a 7 x 23 grid of 161 equal sized rectangles that were placed on the identical positions on each individual image of the 2DE Western blots based on pI and MW. The average density of each rectangle was used to determine whether Sp185/333 proteins with varying pI identified in multiple sea urchins differed in prevalence within a given MW region on a blot. Duplicate samples from the same sea urchin were run and analyzed in parallel to ensure that the observed shifts were not artifacts of protein spreading in 2DE that resulted from sample processing (S1 Protocol; S3 Fig).

## Results

### A subset of native Sp185/333 proteins can be isolated by nickel affinity

**Fig 2:**
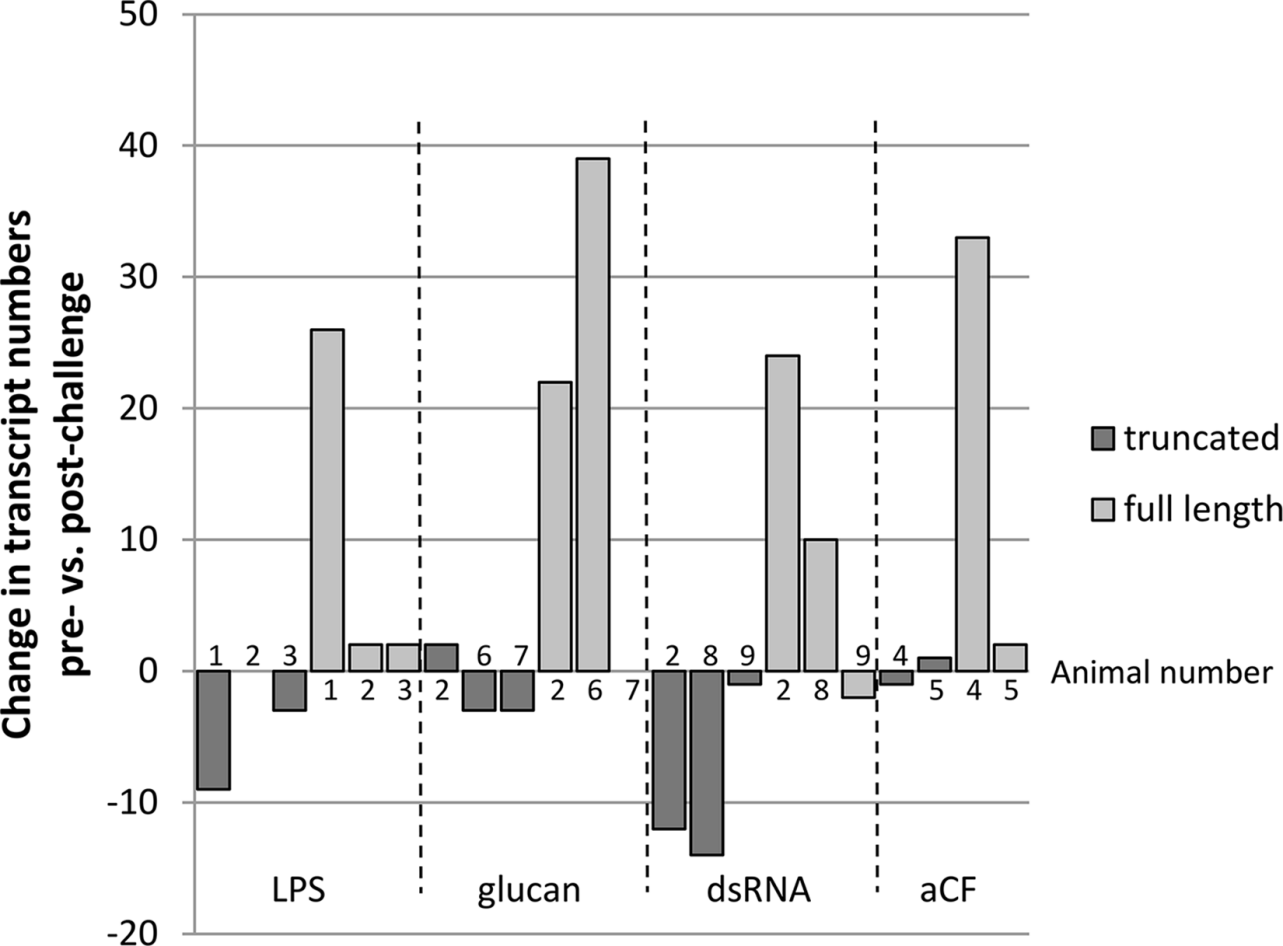
*Sp185/333* mRNA editing decreases in response to immune challenge. *Sp185/333* cDNA sequences reported by Terwilliger et al. [7] were evaluated for the change in the number sequences encoding truncated vs. full-length Sp185/333 proteins post-challenge compared to pre-challenge with LPS, β,1–3,glucan (glucan), double stranded RNA (dsRNA), or artificial coelomic fluid (aCF, buffer, sham injection control). Bars that appear to be missing indicate 0 change. Nine sea urchins (indicated by number) were used in the analysis and animal 2 was evaluated for multiple types of challenges.

Native Sp185/333 proteins are highly diverse yet their overall structure is conserved with regions that are glycine rich and histidine rich (reviewed in [2,3]; Fig 1A). To analyze the diversity of the native Sp185/333 proteins, a method for their isolation from wCF employed histidine affinity for nickel. Animals were immune activated with sets of injections of either heat-killed *Vibrio diazotrophicus*, *E*. *coli*, or LPS to induce or increase the amount of Sp185/333 proteins in the wCF. wCF lysates were loaded onto Ni-affinity columns and Western blots of wash and elution fractions demonstrated that suites of Ni-Sp185/333 proteins could be isolated successfully with this method. Preliminary results of Ni-Sp185/333 proteins from eight of 11 animals showed a range of diversity similar to that observed previously [9,10], yet the suites of proteins isolated from different animals shared a set of common sizes (Fig 1B; Table 2). This suggested that subsets of Sp185/333 proteins with similar histidine signatures were shared among animals and bound well to nickel affinity columns. We expected that the Ni-Sp185/333 proteins would be a subset of the total complement of Sp185/333 proteins in wCF because mRNA editing results in the production of truncated proteins [26], including some with missense sequence [9]. Analysis of *Sp185/333* cDNA sequences reported by Terwilliger et al. [7] and acquired from GenBank indicated decreased numbers of truncated sequences post-challenge (8 of 11 animals), whereas the numbers of full length sequences increased (9 of 11 animals) (Fig 2; and see [2]). Truncated Sp185/333 proteins missing the histidine rich region or full length versions without sufficient numbers of histidines to bind nickel would not be isolated with this approach. This was a possible explanation for the subset of sea urchins that expressed Sp185/333 proteins that could not be isolated by nickel (Table 2). Analysis of Ni-Sp185/333 proteins by Coomassie and silver stained gels showed that additional proteins were present (Fig 1C) and indicated that nickel affinity isolation recovered other wCF proteins in addition to Sp185/333 proteins. Image and densitometry analysis of protein bands on Coomassie stained gels of nickel bound proteins from 11 sea urchins estimated that, on average, only about 20–35% of total protein recovered from each isolation was Sp185/333 proteins (Table 2). However, when proteins isolated by nickel affinity were evaluated with antibodies against two other sea urchin proteins, the complement homologue, SpC3 [27], and the actin binding profilin homologue, SpProfilin [17], these were below the detection level for Western blots (Fig 1C).

**Table 2:** Ni-Sp185/333 proteins isolated from immune activated sea urchins show a wide range of size and variable presence among animals.

Animal	Immune challenge^1^	Ni-Sp185/333 protein sizes (kDa)^2^	Total protein yield % Sp185/333^3^
1	None	~250, 200, 145, 85	nd^4^
5	*Vd*	None	na
6	*Vd*	~230, 200, 140, 82	25 μg/ml; 30%
7	None	~250, 80	21 μg/ml; 45%
8	*Vd*	~85, 50	9 μg/ml; 35%
9	*Vd*	~200, 140, 85	6 μg/ml; 20%
12	*Vd*, *Ec*, L	None	na
13	No	~250, 200, 85, 35	nd
15	*Vd*	~200, 140, 85	4 μg/ml; 28%
17	*Vd*, *Ec*, L	None	na
30	*Vd*	~250, 185, 145, 80	25 μg/ml; 30%

^1^ *Vd*, *Vibrio diazotrophicus* injection (10^4^−10^6^/ml wCF); *Ec*, *E*. *coli* injection (10^4^ to 10^6^/ml wCF); L, LPS injection (1–2 μg/ml wCF).

^2^Protein isolation by nickel affinity followed the protocol recommended by CloneTech.

^3^The percentage of Sp185/333 proteins of the total protein yield was estimated by densitometry of Western blots and corresponding protein gels; see Materials and Methods.

^4^nd, not done; na, not applicable.

Subsequent evaluation of Sp185/333 proteins followed optimized methods for sea urchin cell lysis and 2DE for sea urchin proteins, which took into consideration the high salt concentrations of the wCF (see S1 Protocol; S1, S2 and S3 Figs). To determine whether sea urchin proteins other than the Sp185/333 proteins were isolated by the optimized nickel affinity protocol, we excised spots from two 2DE SDS-PAGE gels (S4 Fig) that were analyzed by ESI-LTQ-MS/MS (MS). Results showed matches to multiple *S*. *purpuratus* proteins per spot indicative of different proteins with the same MW and pI (S2 Table). Although some proteins were localized to at least one or two spots in a given gel, others were identified in multiple spots (S4 Fig; S2 and S3 Tables). The presence of a single protein in multiple spots or different proteins in the same spot was indicative either of isoforms of the same MW or of post-translational modifications to proteins, including phosphorylation (pI change), acetylation (MW change), and glycosylation (pI and MW change). Although Coomassie does not always stain Sp185/333 proteins when present in low concentrations [9], we analyzed a sample of spots present in the Coomassie stained gel that correlated with the locations of Ni-Sp185/333^+^ spots on the Western blot. This association mapping identified five Sp185/333 isoforms in spots positioned within regions of the Western blot that corresponded to Sp185/333^+^ spots (box a; S4B and S4C Fig) whereas none were identified from a region of the gel that did not correspond to a Sp185/333^+^ spot on the Western blot (box b; S4B and S4C Fig). Additional immune related proteins that were identified by MS included thioester containing proteins such as the complement homologue SpC3 (reviewed in [27]), which has opsonin activity [28], complement component C5 and cobra venom factor (both likely matches to SpC3) and a2 macroglobulin [29], which has protease inhibitor activity (S2 Table). Other complement related proteins identified by MS from gels included complement C2 (likely a match to factor B), SpBf that is a homologue of the second component in the alternative pathway (reviewed in [27], and complement related-long precursor, which is speculated to have complement regulatory function [30]. There were multiple matches to scavenger receptor cysteine-rich (SRCR) proteins, which are a diverse set of receptors expressed by coelomocytes [1,31], and homologues of Deleted in Malignant Brain Tumor 1 protein, which is a member of the SRCR protein family and the mammalian homologue functions in innate immunity [32]). Several proteins were identified that have been functionally linked in the clotting reaction including i) amassin and the ii) ammasin-2-like protein noelin [33], which are both members of the olfactomedin domain family (reviewed in [34]), iii) homologues of coagulation factors X and XIII, and iii) arylsulfatase [35]. Other notable matches included melanotransferrin and major yolk protein, which are members of the transferrin family [36] and may be metal ion binding proteins, and the conserved protein DD104 [6] that has unknown functions but has been repeatedly identified in a wide variety of species and is upregulated in immune and stress responses.

To understand why proteins other than the Sp185/333 proteins were isolated by nickel affinity, the FASTA sequences obtained from the MS analysis were scanned for the presence of histidine patches. As expected, the five Sp185/333 isoforms were the only proteins with a patch of more than three histidines that had no other intervening amino acids (S2 Table). Although nickel resin is employed to bind histidine, it also binds other amino acids such as aspartic acid, glycine, lysine, and serine [37] and patches of three or more these amino acids (without intervening amino acids) were identified in the proteins identified by MS (S2 Table). The abundance of these proteins, the patches of the other nickel binding amino acids, as well as the reduced number of imidazole washes in this protocol may explain why proteins without histidine patches were isolated with nickel columns. It is not known whether any of these proteins function as binding partners with the Sp185/333 proteins, however, all had patches of amino acids that likely enabled direct binding to the Ni column.

### There are multiple pI variants of Ni-Sp185/333 proteins within each MW

**Fig 3:**
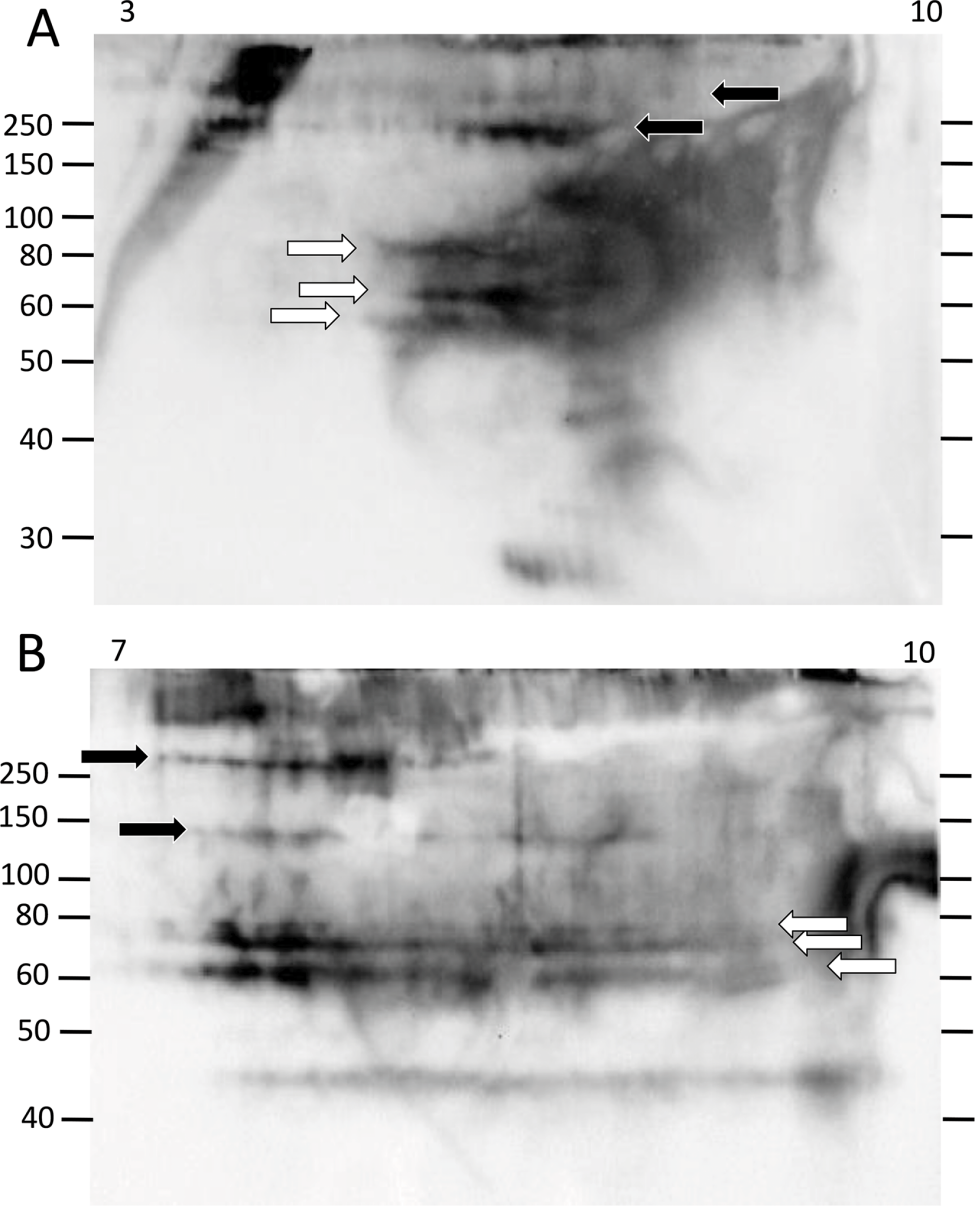
Ni-Sp185/333 proteins of a single MW by 1DE/Western blots resolve to multiple spots and trains in the basic pI range by 2DE/Western blots. The sizes of the Ni-Sp185/333 proteins from sea urchin 101 and that were isolated under optimized protocols (see S1 Protocol) and analyzed by 2DE/Western blot, range in MW from ~40 kDa to over 200 kDa. Most MW sizes are composed of multiple pI variants of which most have a basic pI. (A) A complex of variants with different pI are present within the pI range of 3 to 10, and appear as multiple spots and trains of proteins of ~60 to 80 kDa (white arrows) and >150 kDa (black arrows). (B) The same sample is evaluated in basic pI range of 7 to 10, which is the location on 2DE gels to which most of the Sp185/333 charge variants migrate. Protein trains of similar size and pI as that in A are identified by corresponding arrows.

The limited range of Ni-Sp185/333 protein sizes obtained by 1DE Western blots did not correspond with the wide range of predicted Sp185/333 protein sizes based on coelomocyte cDNA sequences [7,8]. This could be due to only a small subset of Sp185/333 proteins with adequate histidines to enable isolation by nickel affinity, or to multiple isoforms for a given MW, or both. Optimized methods for isolating Ni-Sp185/333 proteins from wCF and evaluating their diversity by 2DE/Western blots, cell lysis and nickel affinity protocols were employed (see S1 Protocol). The Ni-Sp185/333 proteins from sea urchin 101 showed numerous spots and smears such that each MW had Ni-Sp185/333 proteins of varying pI (3 to 10) that were primarily in the physiologically neutral to basic range (Fig 3A). The protein sizes with greatest number of pI variants were generally between 60 to 80 kDa and 125 to >250 kDa. All proteins of these size ranges were likely to be multimers based on predictions of monomer sizes from published results [7,9,10]. When the proteins from sea urchin 101 were evaluated on a pI range of 7 to 10, the pattern of spots for Ni-Sp185/333 proteins of ~60 to 80 kDa appeared as three horizontal lines or trains of proteins (white arrows; Fig 3B). This particular sea urchin also had shorter trains of high MW proteins (>150 kDa) that were present in greater abundance in the basic range than in the acidic range (black arrows; Fig 3B). The prevalence of basic proteins after isolation by nickel affinity was consistent with the positive charge and basic pI for proteins with high histidine content, and also indicated the accessibility of the histidines in Sp185/333 proteins for binding to nickel on the affinity columns. Trains of proteins with the same MW may be indicative of post translational modifications such as phosphorylation or acetylation events that may expand the pI of a given isoform. Alternatively, multiple protein variants of the same MW but with different sequences that impart a range of pI may result in horizontal trains of proteins.

### Ni-Sp185/333 protein repertoires are different among sea urchins prior to immune challenge

**Fig 4:**
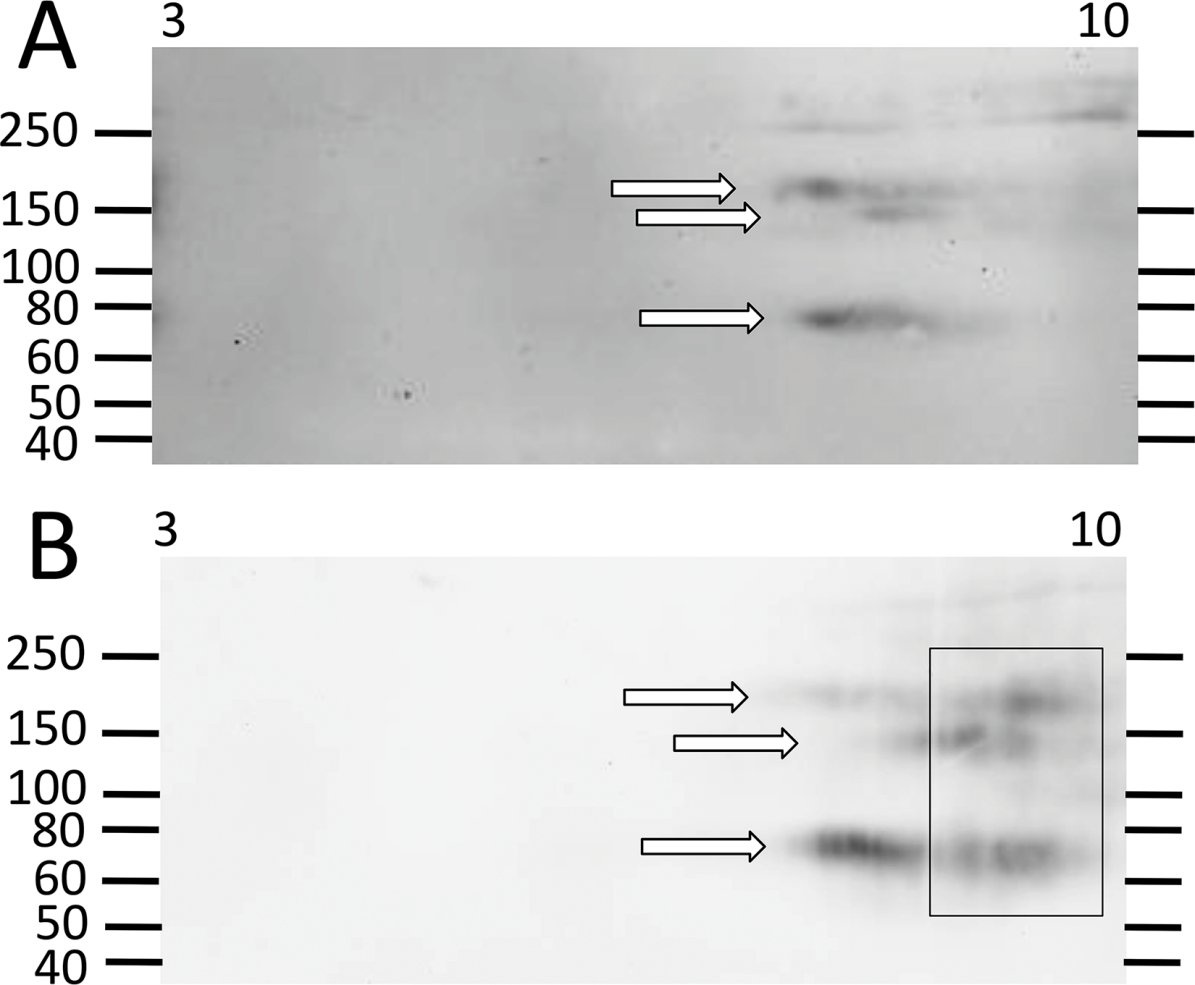
Some sea urchins do not express many Ni-Sp185/333 proteins. A small repertoire of Ni-Sp185/333 proteins is expressed by sea urchin 102 before (A) and after (B) challenge with *V*. *diazotrophicus*. White arrows indicate short trains of proteins with the same MW that are present both before and after challenge. A limited array of more basic proteins appears after challenge (Box in B). These images were cropped on the bottom because they do not show spots of less than 40 kDa.

**Fig 5:**
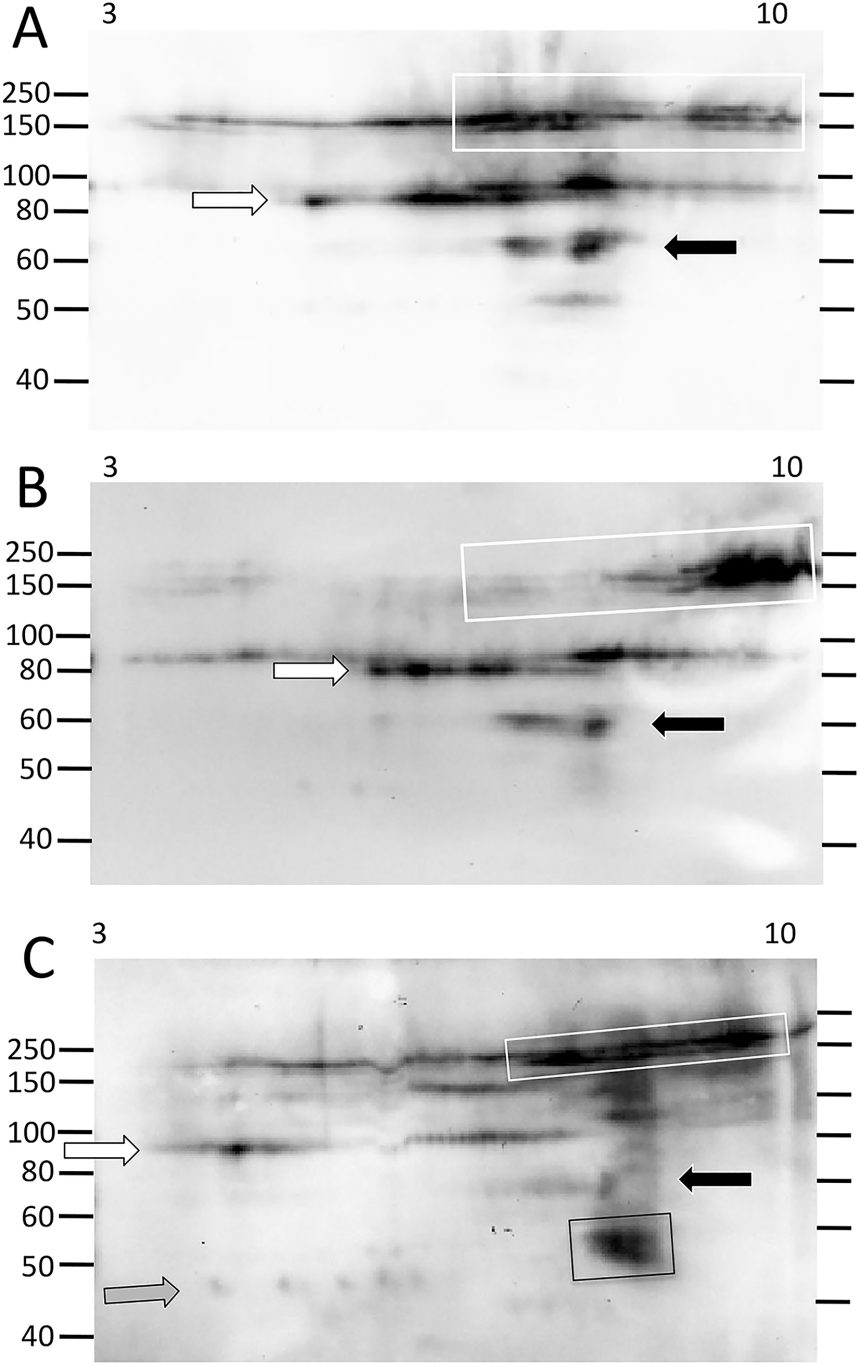
A wide repertoire of Ni-Sp185/333 proteins may be present in some sea urchins both before and after challenge. (A) Sea urchin 103 shows a broad repertoire of Ni-Sp185/333 proteins prior to immune challenge. (B) After the first challenge with *Vibrio diazotrophicus*, the train of ~150 to 250 kDa proteins shifts to much more basic (white box) as does the train of ~90 kDa proteins (white arrow). Proteins of ~60 kDa (black arrow) decrease in intensity compared to those of similar sizes and pI in A. (C) After the second challenge with *V*. *diazotrophicus*, the train of ~150 to 250 kDa proteins is more evenly distributed along the pI gradient (white box), the ~90 kDa proteins extend into the more acidic range (white arrow), and the ~60 kDa proteins decrease in intensity (black arrow). Proteins of new MW/pI appear, which may be monomers (black box, gray arrow). These images were cropped on the bottom because they do not show spots of less than 40 kDa.

The *Sp185/333* genes are expressed by phagocytes from sea urchins and the mRNAs show extensive sequence diversity [7]. Although many of the sequences are quite similar, few share identical sequences among animals. This is in agreement with the finding that there are no genes of identical sequence among animals [2,38]. A limited number of Sp185/333 proteins have been identified from wCF lysates by proteomic approaches [29] and only a few animals have been employed for 2DE/Western blot analyses of Sp185/333 protein arrays in wCF [9]. To expand and improve on these results, we evaluated additional animals prior to immune activation and after challenge with various heat-killed microbes. We removed irrelevant proteins in the wCF by nickel affinity, which improved Ni-Sp185/333 protein detection by Western blot after 2DE separation. In accordance with previous studies using wCF [9], we found that most sea urchins (n = 6) prior to challenge did not consistently express Ni-Sp185/333 proteins. For those animals that did express Ni-Sp185/333 proteins prior to immune challenge, the repertoires were limited compared to animals that were evaluated post challenge (see below). Three of the six animals expressed Sp185/333 proteins prior to challenge that were evident in the wCF by 1DE/Western blot, but these isoforms could not be isolated successfully by nickel affinity (S2C Fig). This result was in agreement with the prevalence of edited *Sp185/333* mRNAs in immunoquiescent animals prior to immune challenge that encoded missense and truncated proteins (Fig 2) and would often lack the histidine rich region and therefore could not be isolated by nickel affinity [7,26]. Three of six sea urchins prior to immune challenge expressed sufficient Ni-Sp185/333 proteins to proceed with 2DE/Western blots. Two animals had fewer than 15 unique MW/pI Ni-Sp185/333 variants (one example is shown; Fig 4A), whereas the third, sea urchin 103, had a wide range of Ni-Sp185/333 proteins (Fig 5A), and may not have been immunoquiescent prior to the start of the experiment. Low or no expression of *Sp185/333* genes and proteins in most of the immunoquiescent sea urchins was consistent with previous reports [6,7,10].

### Ni-Sp185/333 protein repertoires change in response to different immune challenges

#### Individual sea urchins express different repertoires of Ni-Sp185/333 proteins before and after immune challenge

**Fig 6:**
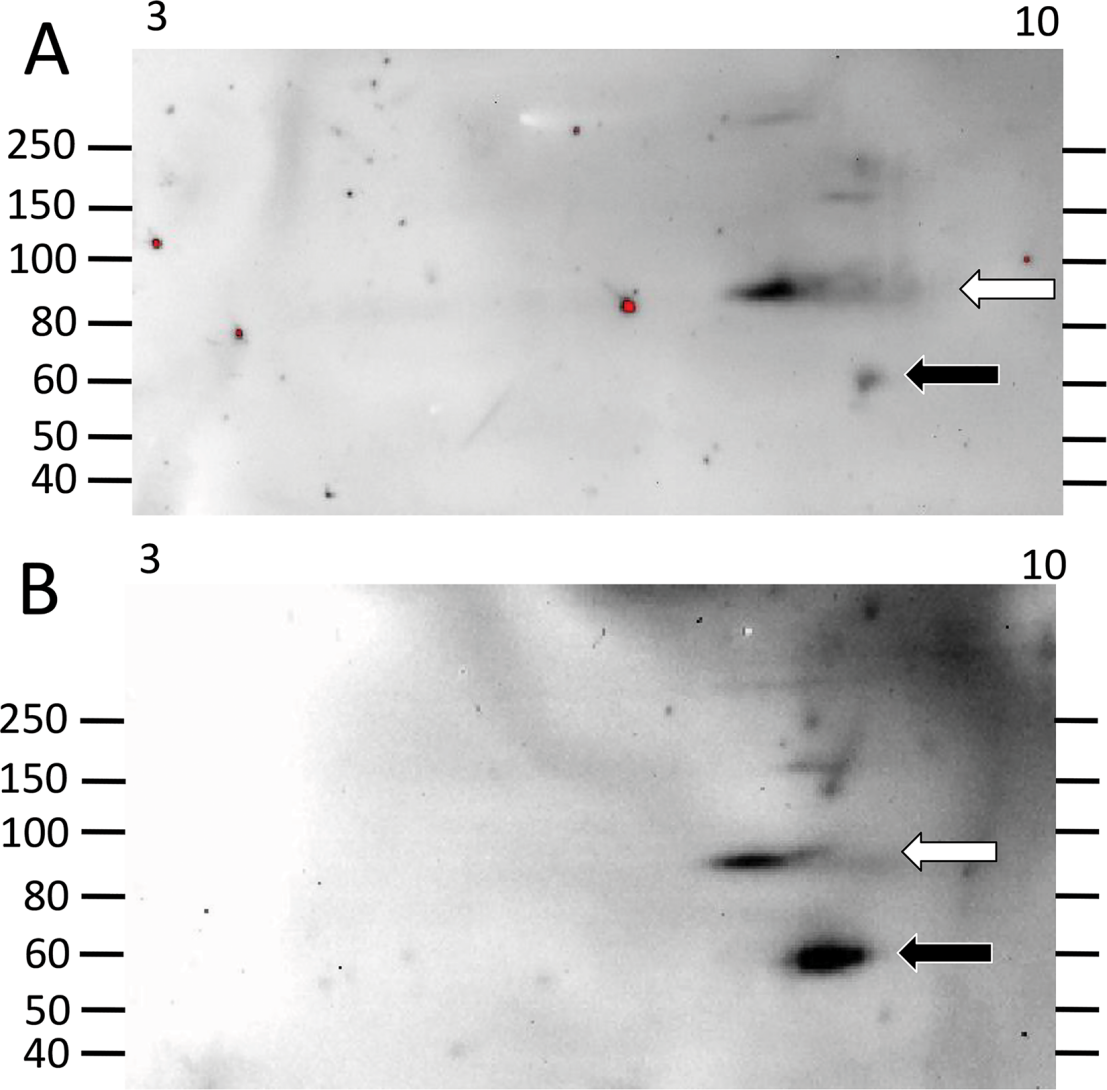
A few Ni-Sp185/333 proteins shift in abundance following immune challenge. In response to immune challenge, sea urchin 104 changes the abundances of some Ni-Sp185/333 proteins before (A) compared to after (B) challenge with *V*. *diazotrophicus*. Although the larger proteins do not change in intensity (white arrow), there is an increase in the size and intensity of the Sp185/333^+^ spot at ~60 kDa and pI ~8 (black arrow) after challenge. These images were cropped on the bottom because they do not show spots of less than 40 kDa.

**Fig 7:**
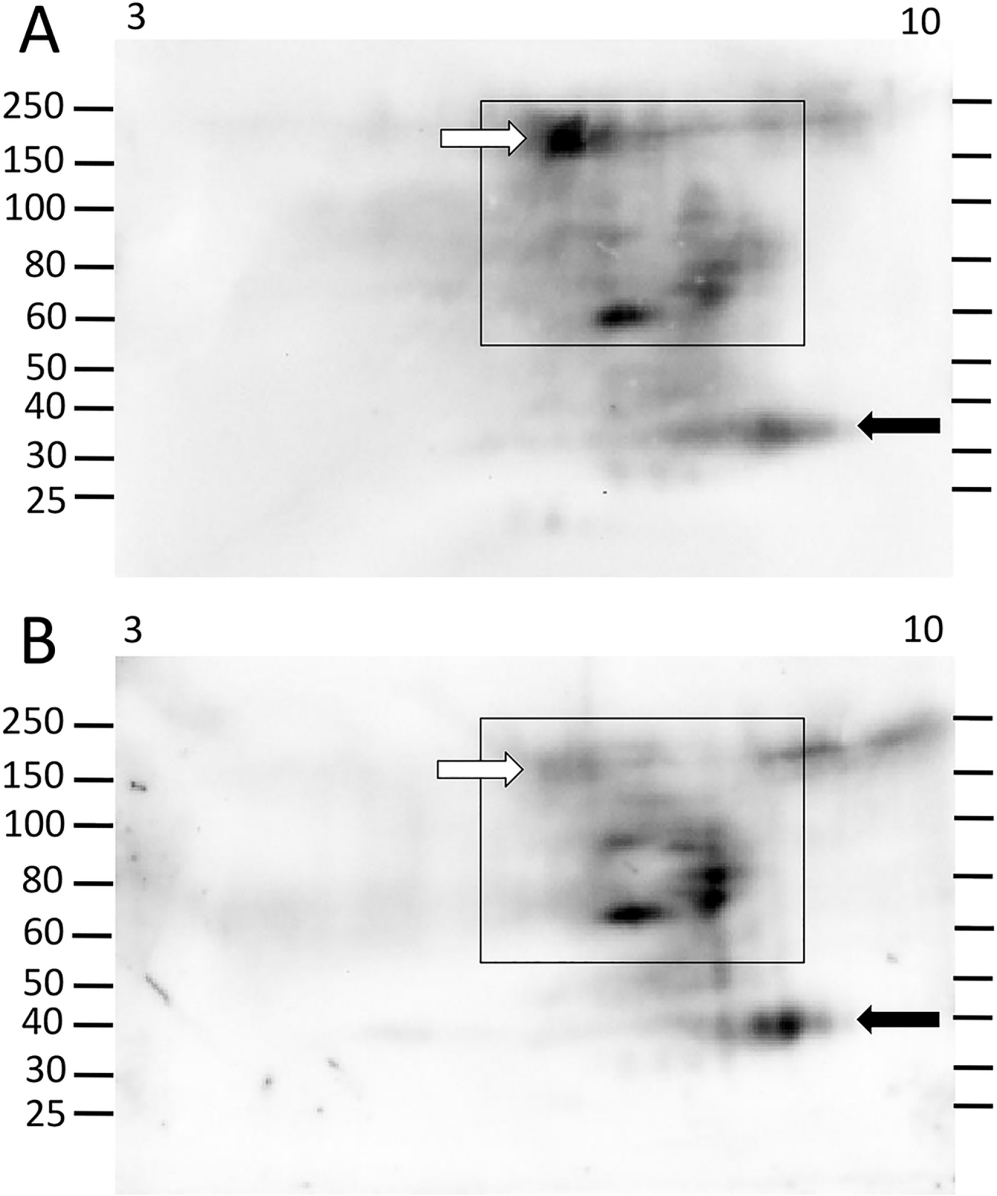
Immune challenge increases the intensity of some Ni-Sp185/333 proteins and decreases the intensity of others. (A) Prior to immune challenge, sea urchin 105 shows a range of Ni-Sp185/333 proteins ranging from ~35 kDa (likely monomers, black arrow) to ~150 to 250 kDa that are all within the pI range of ~7 to 10 (black boxes). (B) After challenge with *V*. *diazotrophicus*, larger MW proteins decrease in intensity (compare white arrows in A and B), whereas the monomers increase in intensity (compare black arrows in A and B).

**Fig 8:**
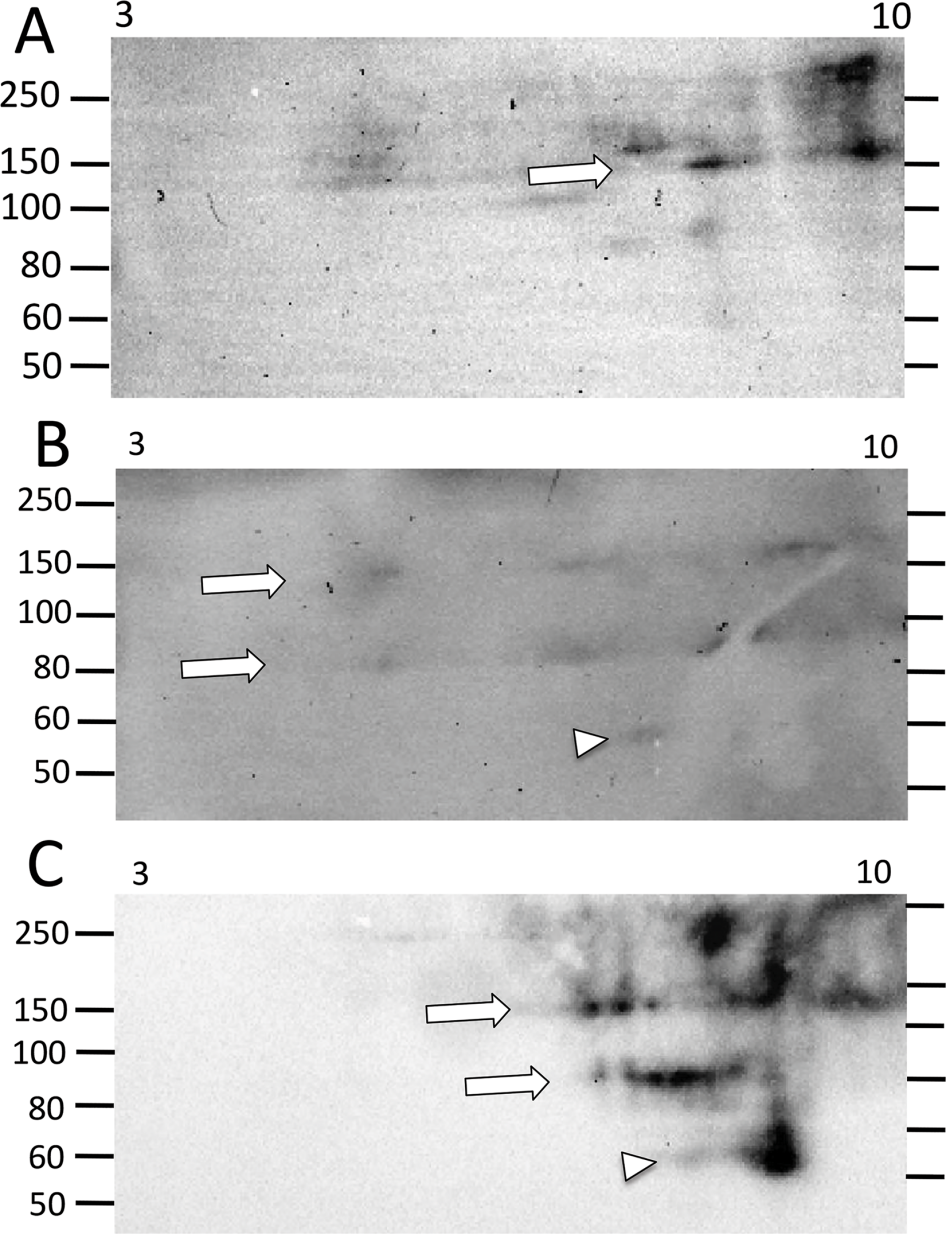
Major changes in the Ni-Sp185/333 protein repertoire may not occur until after the second challenge with *V*. *diazotrophicus*. (A) Sea urchin 106 has a limited array of Ni-Sp185/333 proteins prior to immune challenge, with a few high MW proteins of basic pI (arrow). (B) After the first challenge with *V*. *diazotrophicus*, the Ni-Sp185/333 proteins appear as more acidic (arrows) plus proteins of ~60 kDa appear (arrow head). (C) After the second challenge with *V*. *diazotrophicus*, a significant change in the repertoire of Ni-Sp185/333 proteins shows a shift to more basic pI for both the large MW proteins (arrows), and the ~60 kDa proteins (arrowhead). These images were cropped on the bottom because they do not show spots of less than 50 kDa.

**Fig 9:**
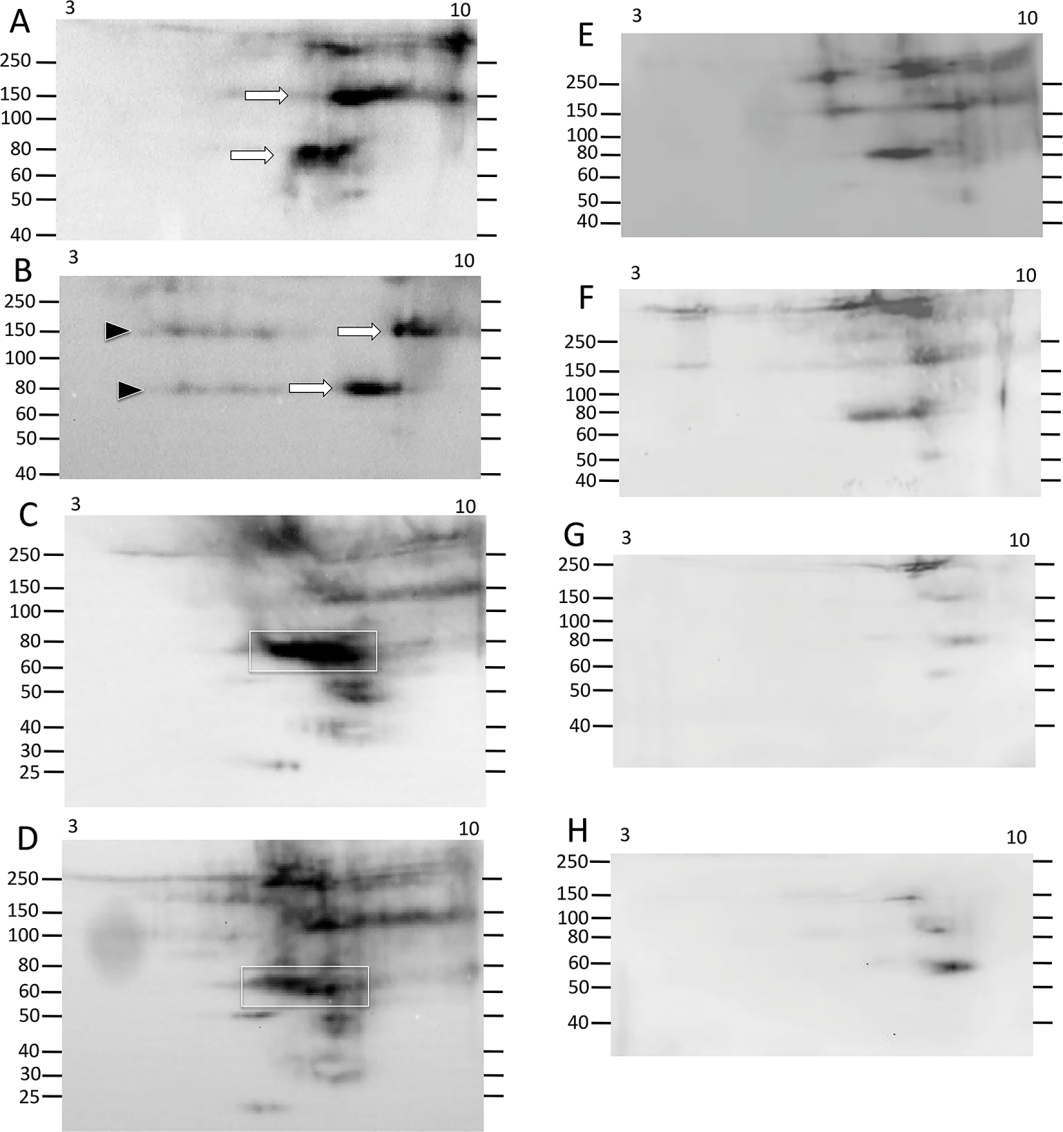
A wide range of changes in the MW/pI of Ni-Sp185/333 proteins can occur over time and in response to multiple challenges with different types of microbes. (A) Prior to immune challenge, sea urchin 107 shows two major trains of Ni-Sp185/333 proteins of ~80 and ~150 kDa (arrows). (B) After the first challenge with *V*. *diazotrophicus*, the majority of the proteins in the trains shift to more basic (arrows), with an expansion of acidic proteins within the same trains (black arrowheads). (C) Prior to challenge with *Bacillus sp*, sea urchin 107 shows a wide repertoire of Sp185/333 proteins, particularly those of ~60 to 80 kDa and pI of ~7 to 8 (white box). (D) After challenge with *Bacillus sp*, there is a slight decrease in the intensity of the Ni-Sp185/333 proteins, particularly those of ~60 to 80 kDa/pI ~6 to 8 (white box). (E) The second challenge with *Bacillus sp* results in a decrease in the intensity of the repertoire of Sp185/333 proteins. (F) The third challenge with *Bacillus sp* further decreases the repertoire and intensity of the Ni-Sp185/333 proteins. (G) Two weeks after challenge with *Bacillus sp* the Ni-Sp185/333 protein repertoire shows a further decrease in the number of spots and their intensity. (H) Subsequent challenge with *V*. *diazotrophicus* does not induce an increase in the Ni-Sp185/333 protein repertoire, but shows further decreases in diversity. Images (A, B, E-H) were cropped at the bottom because they do not show spots of less than 40 kDa.

**Fig 10:**
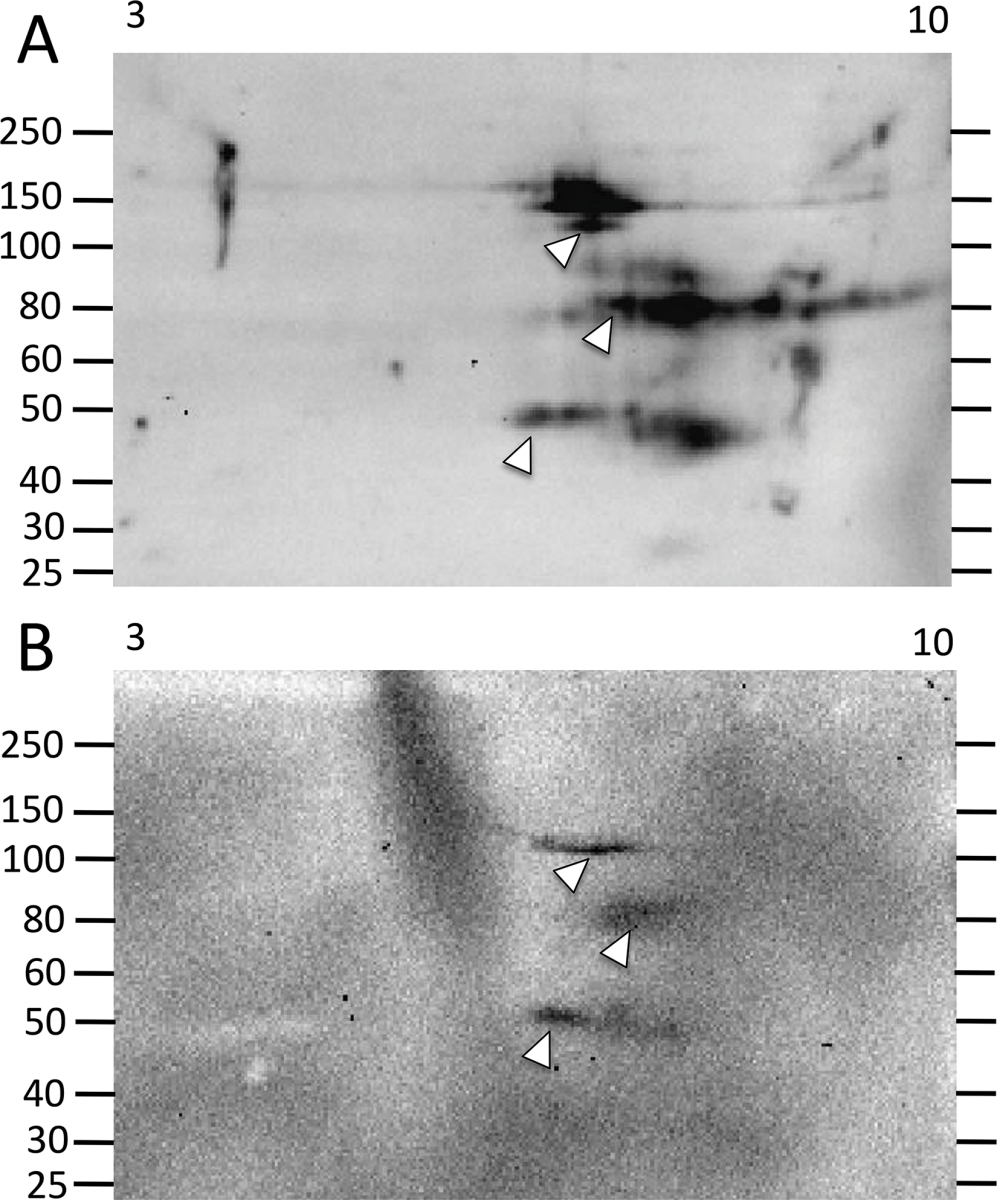
Challenge with *Bacillus sp* does not induce increased expression of Ni-Sp185/333 proteins. (A) Sea urchin 108 shows a repertoire of Ni-Sp185/333 proteins that range in size from ~45 to 200 kDa prior to challenge. (B) After challenge with *Bacillus sp* there is a decrease in the array and intensity of the Ni-Sp185/333 proteins, with only three short trains (arrow heads) matching in size and pI to those observed prior to challenge.

The level of Sp185/333 protein content in the wCF of individual sea urchins changes considerably in response to immune challenge with *Vibrio diazotrophicus* compared to pre-challenge [9,10]. Consequently, when Ni-Sp185/333 proteins were analyzed to compare repertoires before vs. after challenge with *V*. *diazotrophicus*, results from 2DE/Western blots showed that different subsets of Ni-Sp185/333 proteins were expressed both before and after immune challenge among a second set of sea urchins (n = 9) (Figs 4–10). Although most sea urchins did not consistently express Ni-Sp185/333 proteins prior to immune challenges, five of the nine animals that were evaluated expressed Ni-Sp185/333 proteins prior to challenge in at least one wCF sample. Comparisons of Ni-Sp185/333 protein repertoires before vs. after challenge from individual animals showed three categories of outcomes. The first outcome showed arrays of proteins with similar MW and pI before and after immune challenge. This was illustrated by sea urchin 104, which had a small repertoire of Ni-Sp185/333 proteins that did not change MW/pI with regard to challenge with *V*. *diazotrophicus*, however, the intensity of the spots did change (Fig 6). Prior to immune challenge, the majority of the Ni-Sp185/333 proteins were 90 kDa/pI ~7.5 to 9.5 (white arrow; Fig 6A), with a smaller set of spots of 55 to 60 kDa/pI ~8.5 to 9 (black arrow; Fig 6A). Following immune challenge, the proteins at 55 to 60 kDa/pI ~8.5 to 9 increased in intensity (black arrow; Fig 6B), whereas those at 90 kDa/pI ~7.5 to 9.5 did not change (white arrow; Fig 6B). In the second category of outcomes, subsets of protein spots changed abundance within protein arrays, as illustrated by sea urchin 105 (Fig 7). In this example, the repertoire of Ni-Sp185/333 proteins were spread throughout the basic region of the gel (boxes; Fig 7), and showed both increases (black arrows; Fig 7) and decreases (white arrows; Fig 7) in spot intensities at different MW/pI with regard to immune challenge.

The third category of outcomes presented arrays of Ni-Sp185/333 proteins that shifted MW and/or pI after challenge. Several examples in this category were observed including sea urchin 102, which yielded similar numbers of Ni-Sp185/333 MW/pI variants of the same MW, but which shifted after challenge to more basic pI with an increased number of variants (box; Fig 4B). Sea urchin 103 showed a similar shift towards more basic isoforms for proteins of 150 to 250 kDa (white boxes, white arrows; Fig 5A and 5B), but also showed changes in protein abundance (black arrows; Fig 5A and 5B). Sea urchin 106 had unique MW/pI Sp185/333 spots that were present both before and after a single challenge with *V*. *diazotrophicus* that expanded towards acidic pI (arrows; Fig 8A and 8B) in addition to the appearance of ~55 kDa proteins and a slightly basic spot (arrow head; Fig 8B). Sea urchin 107 showed a decrease in the repertoire of Ni-Sp185/333 proteins after the first challenge with *V*. *diazotrophicus* compared to pre-challenge, with shifts towards more basic proteins in the major trains (white arrows; Fig 9A and 9B) and the appearance of more acidic trains after challenge (black arrowheads, Fig 9B). In general, an increase in Ni-Sp185/333 proteins following an initial challenge with *V*. *diazotrophicus* was consistent with reported changes in gene expression [7] in addition to a decrease in edited *Sp185/333* cDNA sequences encoding truncated proteins that was noted in 8 of 11 sea urchins following immune challenge or injury (Fig 2). Overall, a wide range of Ni-Sp185/333 protein repertoires were observed among sea urchins, which illustrated the diversity of the system among animals and the diversity of responses to the same bacterial challenge.

#### Individual sea urchins express different repertoires of Ni-Sp185/333 proteins following repeated challenges with the same bacteria

Given that individual sea urchins expressed different repertoires of Ni-Sp185/333 proteins following an initial set of immune challenges with *V*. *diazotrophicus*, including the appearance of additional MW/pI variants, we next questioned whether individual sea urchins would express the same Ni-Sp185/333 MW/pI variants following a second set of challenges with the same pathogen. Sea urchins were challenged two to four times with three injections per challenge of *V*. *diazotrophicus* and allowed to recover for two weeks between immune challenges. Following each challenge, wCF was collected and analyzed for Ni-Sp185/333 proteins by 2DE/Western blot. Results showed that each sea urchin expressed different repertoires of Ni-Sp185/333 although many MW/pI variants were present consistently following multiple challenges. For example, sea urchin 103 showed a 3.5 fold increase in the number of Ni-Sp185/333 MW/pI protein variants between the first and second challenge with *V*. *diazotrophicus* (Fig 5B and 5C), although proteins within the range of 150 to 250 kDa and pI ~6.5 to 10 were present in both post challenge samples. Following the second challenge with *V*. *diazotrophicus*, the Sp185/333^+^ spots were more intense and were expanded over a wider pI range (white boxes; Fig 5B and 5C). Following the second challenge, the Ni-Sp185/333 protein train at ~75 kDa shifted to a more acidic pI (white arrows; Fig 5B and 5C), and other variants appeared that generated additional trains of proteins of <50 kDa, which were likely monomers (gray arrow; Fig 5C). Furthermore, there was a reduction in intensity of spots at 60 kDa/pI ~8 (black arrows; Fig 5B and 5C) plus the appearance of spots of 45 to 55 kDa and pI ~8 (black box; Fig 5C). In contrast, sea urchin 106 had very different patterns of Ni-Sp185/333 proteins following two immune challenges with *V*. *diazotrophicus* (Fig 8B and 8C). After the initial challenge, two horizontal trains of proteins at 80 kDa and 150 kDa (white arrows; Fig 8B) extended through the basic region of the gel and into the acidic region, plus a single spot at ~55 kDa (white arrowhead; Fig 8B) with a slightly basic pI. Following the second challenge with *V*. *diazotrophicus*, the Ni-Sp185/333 proteins changed significantly such that additional spots appeared, plus the 80 kDa and 150 kDa trains (white arrows; Fig 8C) and the spot at ~55 kDa shifted to more basic and intensified (white arrow head; Fig 8C). Changes in the MW/pI and intensities of the Sp185/333 proteins among sea urchins indicated that different animals responded differently to the same bacteria.

#### Individual sea urchins express different repertoires of Ni-Sp185/333 proteins following immune challenges with different bacteria

Sp185/333 protein expression in sea urchins responding to immune challenges from different pathogen associated microbial patterns (PAMPs; an injection of LPS followed by PGN) showed changes in the patterns of Sp185/333 proteins and/or an increase in the relative quantity of Sp185/333 proteins as evaluated by 1DE/Western blots [9]. To expand on this result using 2DE/Western blots and to make comparisons to changes observed for the Ni-Sp185/333 protein repertoire in response to multiple challenges from *V*. *diazotrophicus* (see previous section), we challenged some of the sea urchins in the second set (n = 7) with additional injections of *V*. *diazotrophicus* followed by *Bacillus sp* (isolate P3.1; Table 1). Surprisingly, only three of seven sea urchins produced sufficient Ni-Sp185/333 proteins for analysis. The initial challenge with *Bacillus sp* for sea urchin 108 resulted in a significantly decreased repertoire of the Ni-Sp185/333 proteins compared to the repertoire observed prior to challenge (Fig 10). This was a reduction from ~45 unique MW/pI variants (Fig 10A) to three short trains of Ni-Sp185/333 proteins of 50, 80, and 100 kDa remaining after challenge (arrowheads; Fig 10). Although sea urchin 107 had many Ni-Sp185/333 protein variants of unique MW/pI present both prior to and following immune challenge with *Bacillus sp*, spot intensities were somewhat decreased in response to *Bacillus sp* (compare white boxes; Fig 9C and 9D). In general, these examples suggested that the repertoire of Ni-Sp185/333 proteins following immune challenge with *Bacillus sp* was different from that following challenge with *V*. *diazotrophicus*, which was best illustrated by sea urchin 107 that received both types of challenges (compare Fig 9B, 9C and 9D). Similar to the responses of sea urchins to immune challenges with *V*. *diazotrophicus*, responses to *Bacillus sp* resulted in changes to the Ni-Sp185/333 protein repertoire that differed among individual sea urchins. In cases where a large repertoire of Ni-Sp185/333 proteins was observed following the initial challenge with either *V*. *diazotrophicus* or *Bacillus sp*, the overall repertoire and abundance of the proteins decreased upon a following challenge with *Bacillus sp* (Figs 1C–9G and 10). This is contrary to results observed for other sea urchins after multiple challenges with *V*. *diazotrophicus* (Figs 5–9A and 9B). Although an initial challenge with *Bacillus sp* led to a decreased Ni-Sp185/333 protein repertoire compared to pre-challenge for sea urchin 107, subsequent immune challenges with *Bacillus sp* resulted in either a change in the distribution of the Ni-Sp185/333 proteins (compare Fig 9C and 9D) or decreased Ni-Sp185/333 protein repertoire (compare Fig 9D, 9E and 9F). When sea urchin 107 was re-challenged with *V*. *diazotrophicus* after responding to *Bacillus sp*, the change in Ni-Sp185/333 protein repertoire before and after challenge was much more modest than the changes observed in response to the initial *V*. *diazotrophicus* challenge (Fig 9G and 9H). Overall, the reduced complexity of the Ni-Sp185/333 protein repertoire in sea urchins responding to *Bacillus sp* was not predicted and unexpected compared to the complex responses to *V*. *diazotrophicus*.

## Discussion

Sea urchins express a wide range of Ni-Sp185/333 protein repertoires composed of many MWs, which resolve into multiple pI variants. Although proteins of many MWs are consistently observed among animals, the range of pI variants for each MW is unique to each sea urchin. Different Ni-Sp185/333 MW/pI variants for individual sea urchins are consistent with different sets and perhaps different numbers of genes in the *Sp185/333* family among animals as suggested by arrays of different *Sp185/333* ^+^ bands on genome blots [8]. Furthermore, *Sp185/333* genes of identical sequence have not been identified among animals [38]. The arrays of Sp185/333 proteins observed on blots are expanded beyond the size of the gene family based on evidence of mRNA editing [26] in addition to putative post-translational modifications such as phosphorylation, glycosylation, or acetylation [7,9]. Sp185/333 isoforms of different pI may interact with one another in different combinations to form complexes that appear as multimers of the same MW but with variable pI. Similarly, isoforms of different pI may combine to form larger protein complexes with a wide range of MW and pI. Although multimerization does not expand the numbers of isoforms that can be produced by a given animal, it may expand the functions of the proteins when acting in complexes. The outcome of the Sp185/333 protein response by sea urchins responding to bacterial challenge is a wide variety of protein arrays that are different among animals and that can be significantly more diverse than the gene family in which they are encoded.

### Multiple Ni-Sp185/333 pI variants are present at each MW

Previous studies have identified Sp185/333 protein arrays of varying MW and pI in wCF from purple sea urchins [9]. However, when wCF is the source for the Sp185/333 proteins, they primarily have an acidic pI range compared to proteins isolated by nickel affinity, which have a more basic pI. This is not unexpected, as an analysis of Sp185/333 proteins from wCF did find small amounts of proteins in the basic range [9] and conversely, some sea urchins have Ni-Sp185/333 proteins in the acidic pI range (shown here). However, Dheilly *et al*. [9] did not investigate the proteins with basic pI as these were a small fraction of the total Sp185/333 proteins detected. This variation in intensity of pI variants that were identified in these two studies is likely due to differences in the approaches for protein isolation and preparation for 2DE. Although both studies evaluated similar amounts of protein for each 2DE/Western blot, the starting point of Ni-Sp185/333 protein enrichment removed many irrelevant proteins from the sample that would be present in wCF, which resulted in a relatively higher concentration of each Ni-Sp185/333 isoform within the total protein sample. The outcome was likely the detection of less abundant, full-length isoforms, although those without nickel affinity were not included in this evaluation.

Most immunoquiescent sea urchins either do not express Sp185/333 proteins [5,7] or lack isoforms that can be isolated by nickel affinity prior to immune challenge. This is consistent with low expression of *Sp185/333* genes or edited *Sp185/333* messages including many with a nonsense mutation at a specific glycine codon that changes the codon to a stop and deletes most of the histidine rich region in many of the truncated proteins [7,26]. These truncated proteins will not be isolated by nickel affinity and are likely to have a more acidic pI. Hence, this is also consistent with the results presented here compared to those reported by Dheilly *et al*. [9]. Following immune challenge, *Sp185/333* mRNA editing decreases [7,26], more of the *Sp185/333* transcripts encode full-length Sp185/333 proteins that include the histidine rich region, and more will be isolated by nickel affinity. Our analysis of the Ni-Sp185/333 protein repertoires observed before and after immune challenge with *V*. *diazotrophicus* is in agreement with previous evaluations of gene expression and mRNA editing predictions showing increased Sp185/333 proteins after challenge [5,7] with either *V*. *diazotrophicus* or LPS [10,39]. However, comparisons among the 2DE/Western blots presented here and those reported by Dheilly *et al*. [9] suggests that the proteins with nickel binding capabilities, are not the majority of the Sp185/333 proteins in sea urchin wCF, and that the repertoire for each animal is likely much more complex than is illustrated here.

### The repertoire of Ni-Sp185/333 proteins is not static for individual sea urchins

The repertoire of Ni-Sp185/333 proteins in individual sea urchins differs following multiple immune challenges with either the same or different bacterial species. Multiple PAMPs, in addition to marine bacteria, upregulate *Sp185/333* gene expression [5–9] and challenges with different PAMPs induce different repertoires of Sp185/333 proteins [9]. The diversity of Ni-Sp185/333 protein MW/pI repertoires following one or two subsequent immune challenges with *V*. *diazotrophicus* or followed by challenges with *Bacillus sp* appears to be a macrocosm of the effects of individual PAMPs. Dheilly *et al*. [9] reported the characteristics of the Sp185/333 repertoire in a sea urchin that was challenged with LPS, and two weeks later was challenged with PGN. The outcome is the expression of many of the same Sp185/333 MW/pI variants following both immune challenges, but detailed analysis of the blots for the two samples show a small number of variants within specific MWs that differ in pI. Here we report that subsequent immune challenges with the same or different bacteria results in some conserved MW/pI variants of Ni-Sp185/333 proteins, but with many more MW/pI variants of Ni-Sp185/333 proteins found in either one sample or the other for individual sea urchins, suggesting a change in Sp185/333 isoform sequence abundances and/or a change in post-translational modifications. Decreases in the the number of spots in a Sp185/333 repertoire may be consistent with a host immune response that has become more focused on the pathogen that has been detected. On the other hand, an apparent decrease in the Ni-Sp185/333 protein repertoire in response to *Bacillus sp* may not infer a decreased or more focused response to this bacterial species compared to *V*. *diazotrophicus*, but may infer a change in the level of Sp185/333 proteins that have nickel binding capabilities. Nevertheless, changes in Ni-Sp185/333 protein repertoire before vs. after immune challenge, points to the possibility that sea urchins may be capable of altering their Sp185/333 protein expression to respond to the class of the infecting pathogen.

### Regulation of *Sp185/333* gene expression; what is the level of complexity?

The complexity of the Sp185/333 protein arrays in individual sea urchins and the varieties of possible changes that are observed over time and in response to pathogen detection raises the question of how this system is coordinated, regulated and controlled. This is particularly interesting given that individual phagocytes express a single *Sp8185/333* gene [15] and presumably produce a single Sp185/333 isoform, although this does not preclude changes to proteins resulting from mRNA editing and post-translational processing. The choice of *Sp185/333* gene that is expressed in a phagocyte, and the coordinated expression of multiple genes among multiple cells, may be orchestrated through PAMP detection by receptors such as sea urchin Toll-like receptors (SpTLRs), which are expressed by coelomocytes [40] among a number of other pathogen recognition receptors (PRRs) [1]. The response by a particular phagocyte may be initiated by a subset of PRRs such as a subset of SpTLRs that show limited expression in individual phagocytes although they are encoded by an expanded family of 253 *SpTLR* genes [40,41]. Because the proteins that likely function in signaling pathways downstream of the SpTLRs, including TIR binding proteins, other signaling proteins and transcription factors, are not encoded by expanded gene families in the *S*. *purpuratus* genome [1,41], the specificity of a phagocyte response to a pathogen may be defined by limited number of different SpTLRs and/or their combinatorial expression [40]. This model predicts that expression of each *Sp185/333* gene may be regulated by i) the set of SpTLRs that are expressed on a phagocyte, ii) the type of pathogen or PAMP to which it can bind to initiate signaling, and iii) the sequences of the regions that regulate expression of the *Sp185/333* genes. Although these speculations are grounds for future work, the outcome of the Sp185/333 protein expression system appears to be highly responsive and flexible, and produces wide ranges of highly variable anti-pathogen protein arrays that may interact quickly and efficiently with the plethora of microbes in the marine environment.

## Supporting Information

S1 Fig Lysis buffer optimization. (A) Lysis buffer selection. A representative Western blot shows the detection of Sp185/333 proteins isolated after treatment of wCF by snap-freeze-thaw or lysis buffer S or C (see Table 1). (B) Detergent selection. A representative Western blot shows the detection of Sp185/333 proteins isolated from two sea urchins after wCF treatment with different detergents; 1% CHAPS (C), 1% sarkosyl (S), 1% Triton-X100 (T), and a mixture of all three (M).
(TIF)

S2 Fig Optimization of binding and eluting Sp185/333 proteins on nickel affinity columns and column fraction evaluation by Western blot for Ni-Sp185/333 proteins. Whole coelomic fluid (wCF) lysate is used for comparisons to the wash and elution fractions. Note that not all Sp185/333 proteins bind nickel; some pass through the column, and are present in the flow through (FT). (A) Manufacturer’s protocol (ClonTech) for nickel isolation results in most of the Ni-Sp185/333 proteins appearing in the first wash fraction, which uses a wash buffer with 20 mM imidazole (wash 1.1). Additional Ni-Sp185/333 proteins are eluted from the resin upon treatment with SDS-lysis buffer at 95°C for 5 min. (B) Optimized nickel isolation. The optimized protocol elutes Sp185/333 proteins that do not bind nickel strongly in the wash buffer fractions (10 mM imidazole). Those that bind well to nickel remain on the column and are collected in the elution buffer containing 300 mM imidazole (elution fractions 1 and 2). No residual Ni-Sp185/333 proteins are eluted from the resin. (C) Prior to immune challenge, many sea urchins do not express Sp185/333 proteins that can be isolated by nickel affinity. Elution from a nickel affinity column of wCF from sea urchin 13 and two other sea urchins (not shown) prior to immune challenge do not yield sufficient Ni-Sp185/333 proteins for further analysis.
(TIF)

S3 Fig Duplicate sample preparation results in identical 2DE/Western blots. (A, B) Sea urchin 110 was sacrificed and total wCF was collected, split into two samples, and processed in parallel for 2DE/Western blots. (C, D) An aliquot of wCF from sea urchin 106 after immune challenge with *V*. *diazotrophicus* was split into two samples and subjected to isoelectric focusing on separate occasions. Samples were passed through a nickel column and proteins were separated by 2DE and evaluated by Western blots with the mixture of the anti-Sp185/333 sera. Blots of proteins from the same animal show spots in the same positions.
(TIF)

S4 Fig Duplicate 2DE gels show the same arrays of nickel isolated proteins. Coomassie staining of 2DE gels of nickel isolated proteins of wCF from animal 7 (A) and 9 (B) shows predominantly basic proteins, consistent with a large number of (positively charged) histidines. Numbers indicate spots excised for mass spectrometric analysis of two animals (see S2 and S3 Tables). Western blot analysis of a sample from animal 9 (C) run in parallel confirms that the Ni-Sp185/333 proteins are also in the basic region of the IEF strip. Stained spots excised for mass spectrometric analysis (box a) is in a region of the gel where the Western blot shows a large amount of Sp185/333 proteins. Box b indicates the spots that were evaluated to ensure complete protein focusing. This region of the gel does not include a large amount of Sp185/333 proteins.
(TIF)

S1 Protocol Protocol Optimization for Nickel binding/elution and 2DE/Western blots. (DOCX)

S1 Table Components of lysis buffers S and C. (DOCX)

S2 Table Unique proteins identified in each spot from 2DE of nickel isolated samples from two sea urchins (DOCX)

S3 Table Proteome Discoverer Results (XLSX)
