# Supplementary figures and images for: Extraordinary Diversity of Immune Response Proteins among Sea Urchins: Nickel-Isolated Sp185/333 Proteins Show Broad Variations in Size and Charge
Source: PLoS One. 2015 Sep 25;10(9):e0138892. doi: 10.1371/journal.pone.0138892 (PMC4583492; doi:10.1371/journal.pone.0138892)

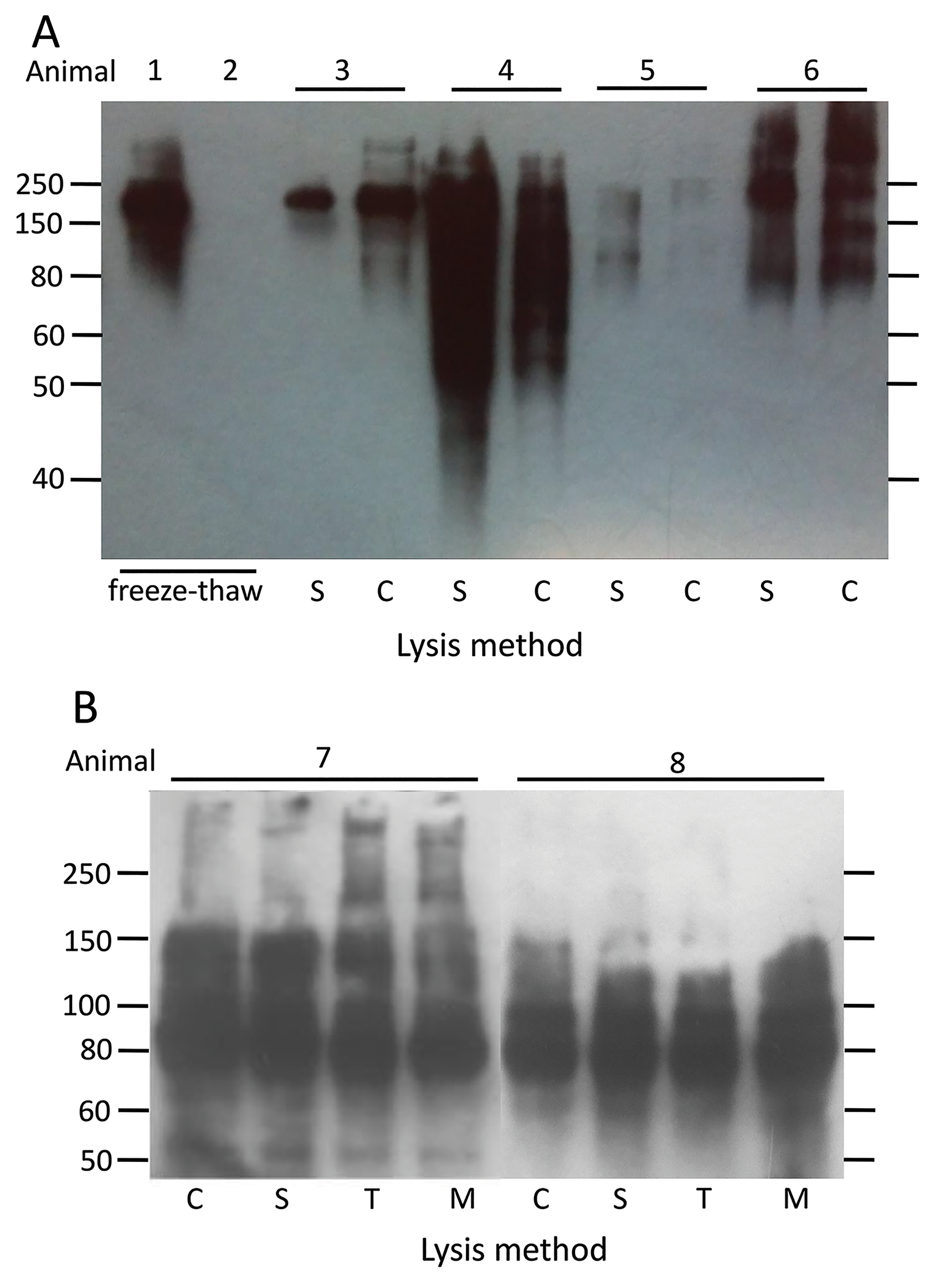

Supplement: S1 Fig — (A) Lysis buffer selection. A representative Western blot shows the detection of Sp185/333 proteins isolated after treatment of wCF by snap-freeze-thaw or lysis buffer S or C (see Table 1). (B) Detergent selection. A representative Western blot shows the detection of Sp185/333 proteins isolated from two sea urchins after wCF treatment with different detergents; 1% CHAPS (C), 1% sarkosyl (S), 1% Triton-X100 (T), and a mixture of all three (M). (TIF) [file pone.0138892.s001.tif]

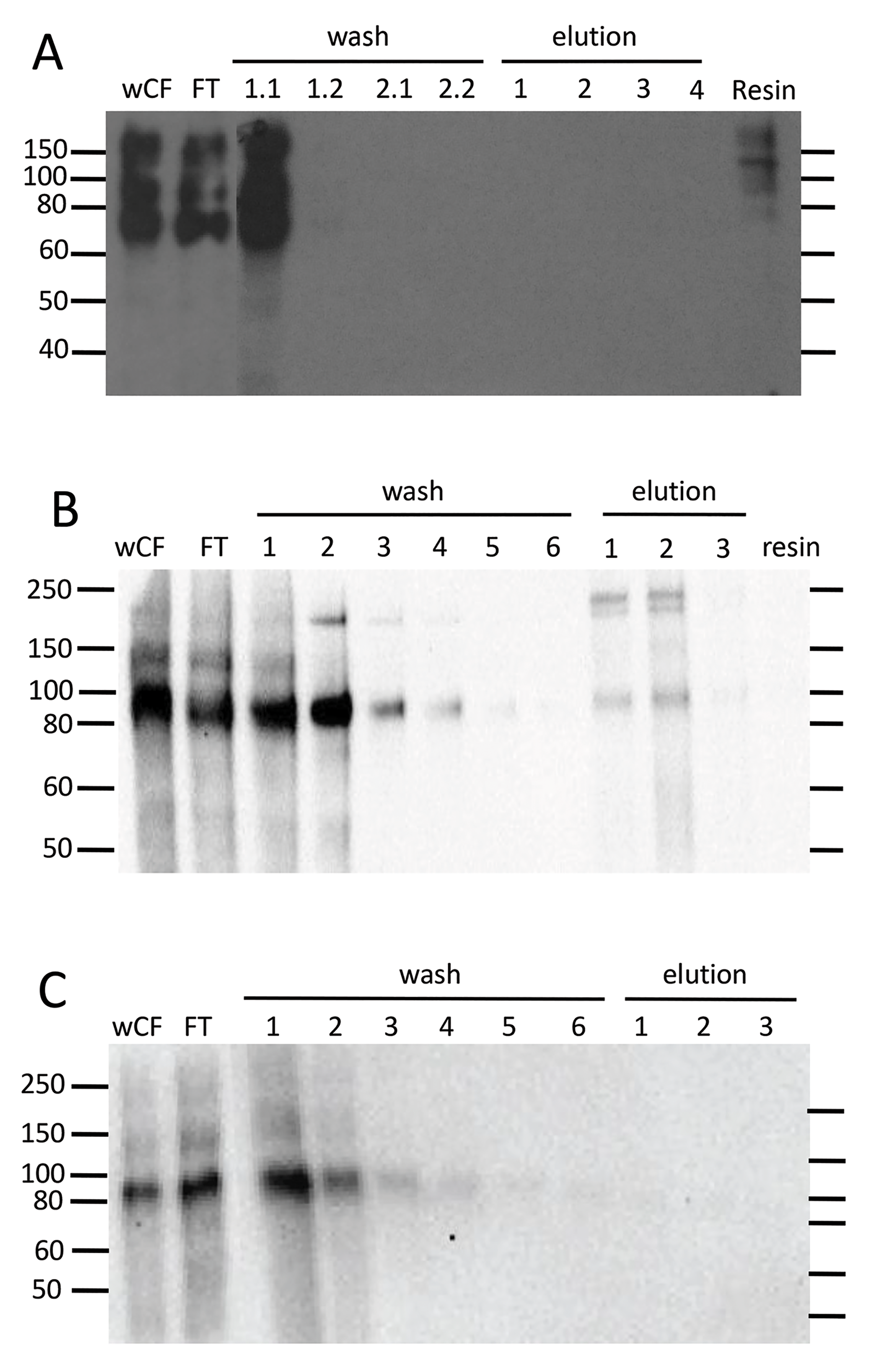

Supplement: S2 Fig — Whole coelomic fluid (wCF) lysate is used for comparisons to the wash and elution fractions. Note that not all Sp185/333 proteins bind nickel; some pass through the column, and are present in the flow through (FT). (A) Manufacturer’s protocol (ClonTech) for nickel isolation results in most of the Ni-Sp185/333 proteins appearing in the first wash fraction, which uses a wash buffer with 20 mM imidazole (wash 1.1). Additional Ni-Sp185/333 proteins are eluted from the resin upon treatment with SDS-lysis buffer at 95°C for 5 min. (B) Optimized nickel isolation. The optimized protocol elutes Sp185/333 proteins that do not bind nickel strongly in the wash buffer fractions (10 mM imidazole). Those that bind well to nickel remain on the column and are collected in the elution buffer containing 300 mM imidazole (elution fractions 1 and 2). No residual Ni-Sp185/333 proteins are eluted from the resin. (C) Prior to immune challenge, many sea urchins do not express Sp185/333 proteins that can be isolated by nickel affinity. Elution from a nickel affinity column of wCF from sea urchin 13 and two other sea urchins (not shown) prior to immune challenge do not yield sufficient Ni-Sp185/333 proteins for further analysis. (TIF) [file pone.0138892.s002.tif]

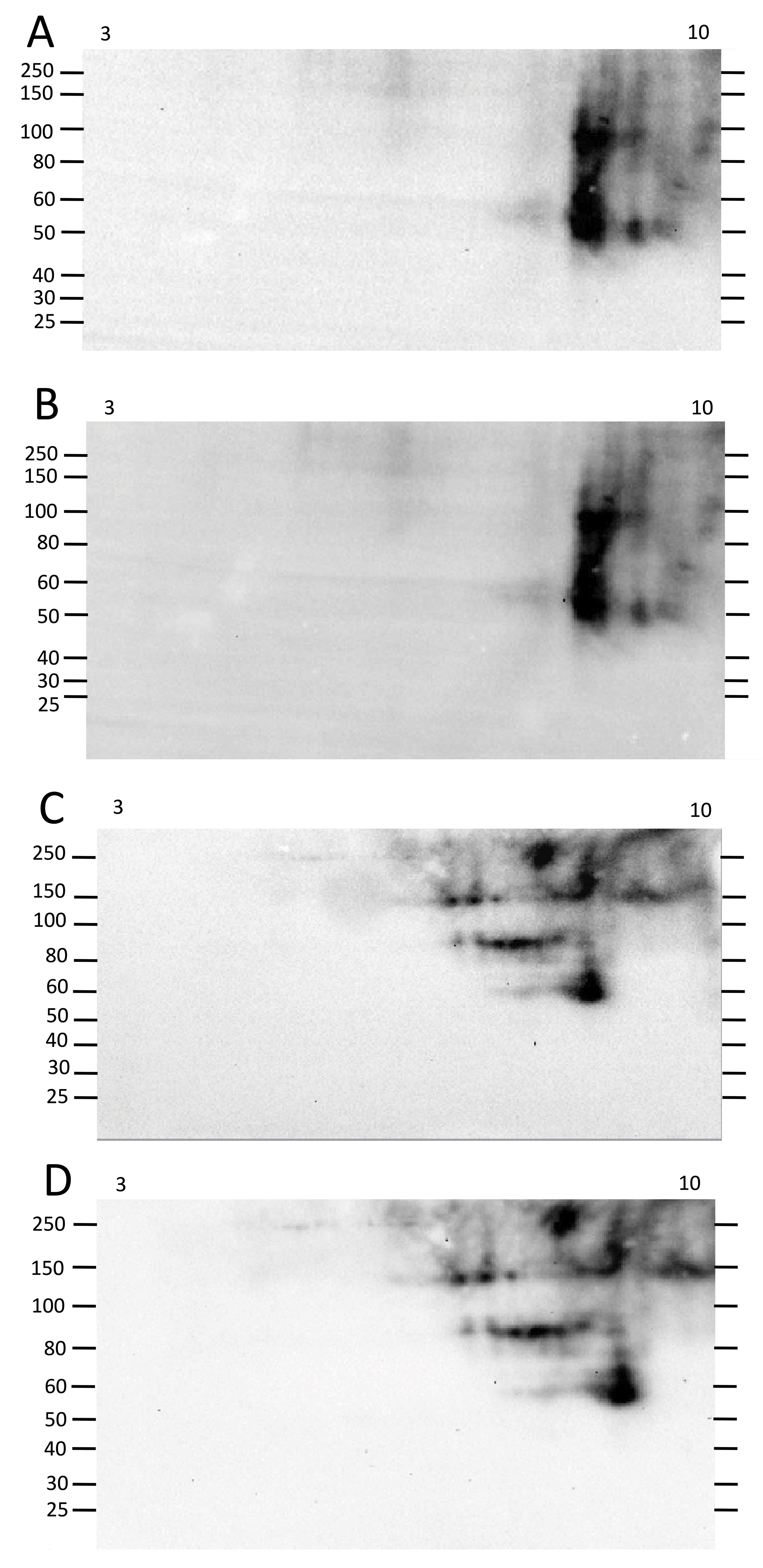

Supplement: S3 Fig — (A, B) Sea urchin 110 was sacrificed and total wCF was collected, split into two samples, and processed in parallel for 2DE/Western blots. (C, D) An aliquot of wCF from sea urchin 106 after immune challenge with V. diazotrophicus was split into two samples and subjected to isoelectric focusing on separate occasions. Samples were passed through a nickel column and proteins were separated by 2DE and evaluated by Western blots with the mixture of the anti-Sp185/333 sera. Blots of proteins from the same animal show spots in the same positions. (TIF) [file pone.0138892.s003.tif]

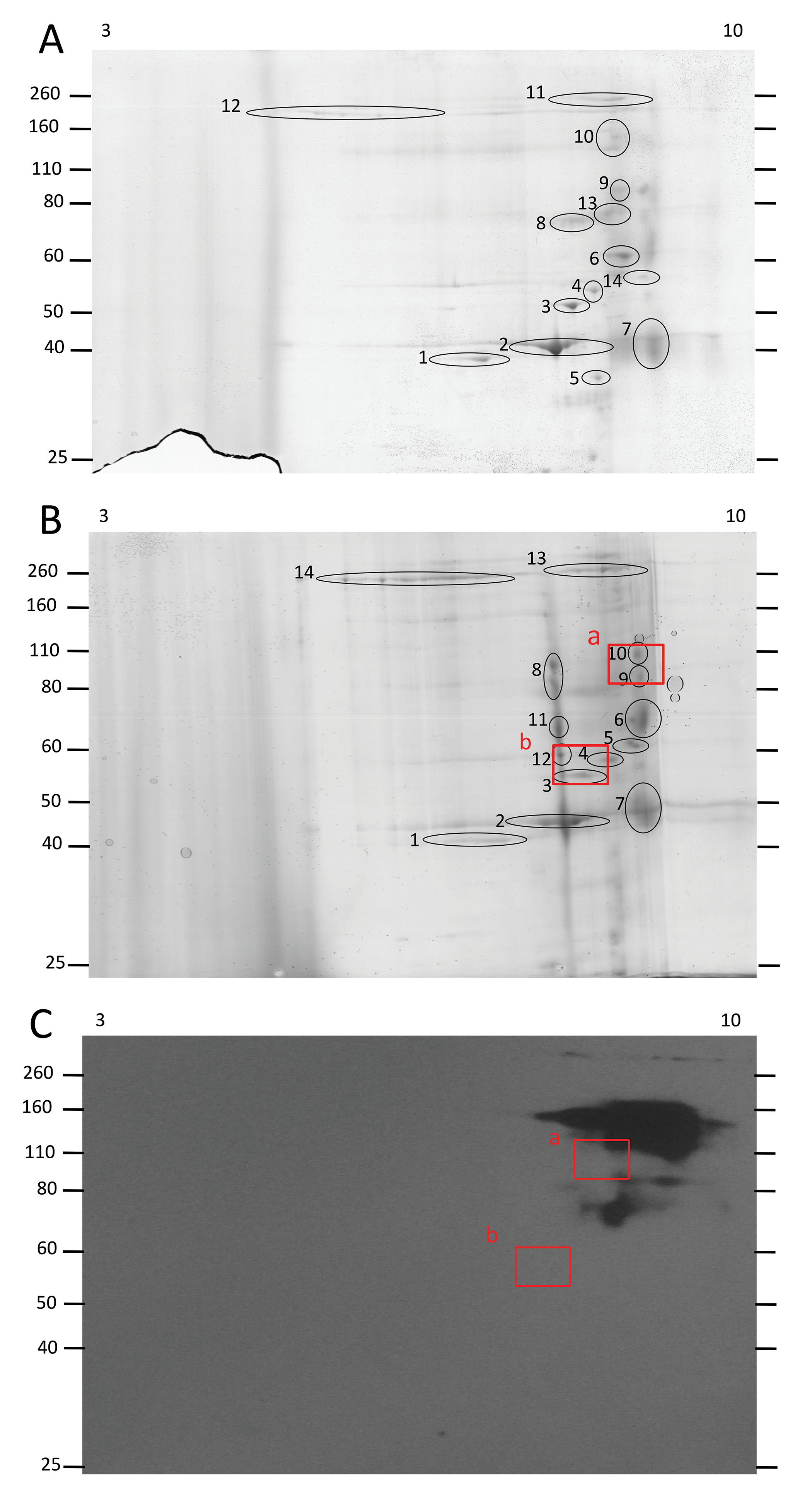

Supplement: S4 Fig — Coomassie staining of 2DE gels of nickel isolated proteins of wCF from animal 7 (A) and 9 (B) shows predominantly basic proteins, consistent with a large number of (positively charged) histidines. Numbers indicate spots excised for mass spectrometric analysis of two animals (see S2 and S3 Tables). Western blot analysis of a sample from animal 9 (C) run in parallel confirms that the Ni-Sp185/333 proteins are also in the basic region of the IEF strip. Stained spots excised for mass spectrometric analysis (box a) is in a region of the gel where the Western blot shows a large amount of Sp185/333 proteins. Box b indicates the spots that were evaluated to ensure complete protein focusing. This region of the gel does not include a large amount of Sp185/333 proteins. (TIF) [file pone.0138892.s004.tif]
