# Supplementary material for: Extraordinary Diversity of Immune Response Proteins among Sea Urchins: Nickel-Isolated Sp185/333 Proteins Show Broad Variations in Size and Charge
Source: PLoS One. 2015 Sep 25;10(9):e0138892. doi: 10.1371/journal.pone.0138892 (PMC4583492; doi:10.1371/journal.pone.0138892)
Supplement: S1 Protocol — (DOCX) [file pone.0138892.s005.docx]

Supporting Information: Protocol Optimization for Nickel binding/elution and 2DE/Western blots

**Diversity of Immune Response Proteins among Sea Urchins: Nickel-isolated Sp185/333 Proteins show Broad Ranges of Size and Charge**

Lauren S. Sherman^1,#a^, Catherine S. Schrankel^1,#b^, Kristy J. Brown^2^, L. Courtney Smith^1^

Author Affiliations

^1^Department of Biological Sciences, George Washington University, Washington DC, United States of America

^2^Center for Genetic Medicine Research, Children’s National Medical Center, Washington, DC, United States of America

^#a^Current Address: Rutgers Biomedical and Health Sciences, Graduate School of Biomedical Sciences, Rutgers University, Newark, New Jersey, United States of America

^#b^Current Address: Sunnybrook Health Sciences Centre and Department of Immunology, University of Toronto, Toronto, Ontario, Canada

**Protocol optimization**

The predicted prevalence of histidines near the C terminal region of the Sp185/333 proteins suggested that they could be used to isolate native proteins on nickel columns. Preliminary results from one dimensional electrophoresis (1DE) followed by Western blots of Sp185/333 proteins isolated by nickel column purification show major bands at ~80 to 90 kDa, ~140 kDa, 200 to 220 kDa, and ~250 kDa (see main paper). To evaluate the proteins that appeared by 1DE, maximal amounts of proteins had to be isolated from whole coelomic fluid (wCF), and methods for two dimensional gel electrophoresis (2DE) and Western blot analysis required optimization. We describe here, approaches to optimize these methods which were subsequently used to generate the data for the results reported here.

wCF cell lysis optimization Prior to the analysis of Sp185/333 protein diversity among individual sea urchins, several protein isolation methods were evaluated to ensure that the maximum amount and variety of proteins were collected. wCF from immune challenged animals was collected and a cocktail of ice cold protease inhibitors was immediately added to the wCF to prevent protein degradation (10 mM Benzamidine, 1mM phenylmethanesulfonylfluoride, 50 μM Na_2_HPO_4_, 10 μl Protease Inhibitor Cocktail [Sigma-Aldrich]/ml wCF). The coelomocytes were lysed by sonication in either lysis buffer S or C (S1 Table), or by snap freezing followed by thawing in the presence of 1% sarkosyl to lyse the coelomocytes. Lysates were centrifuged to remove particulates, the proteins in the supernatant were separated by 1DE and analyzed by Western blot to determine the number of MW variants of Sp185/333 proteins in the samples. Results showed that neither lysis buffer nor the freeze/thaw treatment worked optimally to lyse the cells for isolating Sp185/333 proteins from wCF, and that in some cases, protein degradation was evident (S1 Fig.). Lysis buffers S and C showed different patterns of Sp185/333 protein bands for samples from the same animal (animals 3-6; S1A Fig.). In some animals, buffer S resulted in more bands than buffer C (animals 4, 5), while for other animals the reverse result was obtained (animals 3, 6). When bands of the same MW were observed for both lysis buffers for a given animal, sometimes buffer S produced stronger bands (animal 4; S1A Fig.) while other times buffer B produced stronger bands (animal 3). Interestingly, some animals showed a mix of patterns. For example, animal 6 had a stronger band of ~200 kDa with buffer S, and a stronger band at ~80 kDa with buffer C. Snap-freeze-thawing produced bands of different MWs in two different animals, as well as compared to the lysis buffers (S1A Fig.).

The effects of detergents on Sp185/333 protein isolation from two animals were also investigated. wCF was collected and coelomocytes were lysed with different detergents in the presence of protease inhibitors (S1B Fig.). Four lysis buffers were employed followed by sonication: 1% CHAPS, 1% sarkosyl, and 1% Triton-X100, as well as a cocktail of the three. Lysates from two sea urchins were evaluated by Western blot and results indicated that the cocktail of all three detergents was optimal for isolating Sp185/333 proteins from wCF because it yielded the maximum number of protein bands (S1B Fig.). Ultimately, a detergent cocktail of 1% CHAPS and 1% sarkosyl was chosen as the ionic nature of Triton-X100 created problems in downstream applications.

Nickel binding and elution optimization The predicted structure of most Sp185/333 proteins shows a significant prevalence of histidines in the C terminal region [1,2], which predicts that they can be isolated on Ni-His60 (ClonTech) nickel columns based on the affinity between histidine and nickel. We thus sought to optimize the commercial nickel affinity isolation protocol that is normally employed for isolating recombinant proteins with histidine tags. When the manufacturer’s instructions were used, the majority of the Sp185/333 proteins were eluted by the wash buffer containing 20 mM imidazole in the initial wash fraction (S2A Fig.). Furthermore, some Sp185/333 proteins bound tightly to the nickel resin and were not eluted with the highest concentration of imidazole (S2A Fig.). The protocol was modified to i) reduce the imidazole concentration to 10 mM in the wash buffer, ii) delete the 40 mM imidazole wash step, and iii) increase the imidazole concentration in the elution buffer to 300 mM (S2B Fig.). This resulted in some Sp185/333 protein loss in the washes prior to elution, but a majority of the proteins were collected in the first 300 mM elution fractions (S2B Fig.). After the Sp185/333 proteins were eluted, no additional Sp185/333 proteins were released from the resin upon treatment with SDS-lysis buffer at 95°C for 5 min.

Although the protocol for isolating Sp185/333 proteins by nickel affinity from immune activated sea urchins was optimized, prior to challenge most sea urchins do not express many *Sp185/333* genes [1,3], which suggested low levels of Sp185/333 proteins in the wCF. However, when Sp185/333 proteins were isolated by nickel affinity for six sea urchins prior to immune challenge, all had Sp185/333 proteins in the wCF, but three had Sp185/333 proteins that could not be isolated by nickel affinity (S2C Fig.). This outcome was consistent with the prevalence of truncated and missense Sp185/333 proteins prior to challenge that would be missing the histidine rich region [1,4,5] (see Fig 1A).

2DE optimization 2DE/Western blot analysis was used to evaluate the complexity of the Ni-Sp185/333 protein arrays among different sea urchins. Initially, passive rehydration was used to introduce the nickel isolated proteins into the IEF strip, however, the proteins did not migrate well onto the strip with this approach, so a small electrical current (50 V) was applied. The high electrical resistance of the nickel isolated proteins made the initial stages of focusing difficult. To reduce this problem, the incremental voltage steps that are often used for IEF were removed and the voltage was set at 8,000 V for 35,000 V-hrs at 20°C. These changes resulted in optimal protein focusing for the samples. If erratic voltage was noted, a wick was inserted to contact the IEF strip to remove some of the remaining salt, which decreased the resistance of the strip. To identify possible protein isolation and IEF artifacts, duplicate protein samples from individual sea urchins were prepared in parallel to determine whether the preparation method resulted in the same spots in MW or pI of the Ni-Sp185/333 proteins (S3A and S3B Fig.). Duplicate samples were also processed for 2DE Western blots on separate occasions to ensure that freeze-thaw cycles from storage and focusing conditions were not a contributing factor to the observed diversity of protein repertoires (S3D and S3C Fig.). Separations for each of these replicates were very similar, showing spots in the basic range of the gel and with the same MW, and suggesting that the diversity observed for the Sp185/333 proteins was not altered or introduced by technical artifacts.

**References**

1. Terwilliger DP, Buckley KM, Brockton V, Ritter NJ, Smith LC (2007) Distinctive expression patterns of *185/333* genes in the purple sea urchin, *Strongylocentrotus purpuratus*: an unexpectedly diverse family of transcripts in response to LPS, beta-1,3-glucan, and dsRNA. BMC Molecular Biology 8: 16.

2. Terwilliger DP, Buckley KM, Mehta D, Moorjani PG, Smith LC (2006) Unexpected diversity displayed in cDNAs expressed by the immune cells of the purple sea urchin, *Strongylocentrotus purpuratus*. Physiological Genomics 26: 134-144.

3. Nair SV, Del Valle H, Gross PS, Terwilliger DP, Smith LC (2005) Macroarray analysis of coelomocyte gene expression in response to LPS in the sea urchin. Identification of unexpected immune diversity in an invertebrate. Physiological Genomics 22: 33-47.

4. Dheilly NM, Nair SV, Smith LC, Raftos DA (2009) Highly variable immune-response proteins (185/333) from the sea urchin *Strongylocentrotus purpuratus*: Proteomic analysis identifies diversity within and between individuals. Journal of Immunology 182: 2203-2212.

5. Buckley KM, Terwilliger D, Smith LC (2008) Sequence variations in *185/333* messages from the purple sea urchin suggest posttranscriptional modifications to increase immune diversity. The Journal of Immunology 181: 8585-8594.
