# Supplementary material for: Extraordinary Diversity of Immune Response Proteins among Sea Urchins: Nickel-Isolated Sp185/333 Proteins Show Broad Variations in Size and Charge
Source: PLoS One. 2015 Sep 25;10(9):e0138892. doi: 10.1371/journal.pone.0138892 (PMC4583492; doi:10.1371/journal.pone.0138892)
Supplement: S1 Table — (DOCX) [file pone.0138892.s006.docx]

**S1 Table**. Components of lysis buffers S and C

| **Lysis Buffer S** | **Lysis Buffer C** |
| --- | --- |
| 1% Sarkosyl | 1% CHAPS |
|  | 7M Urea |
|  | 2M Thiourea |
|  | 40mM Tris-Base, pH 8.8 |
|  | 10mM DTT |
