# Supplementary material for: Extraordinary Diversity of Immune Response Proteins among Sea Urchins: Nickel-Isolated Sp185/333 Proteins Show Broad Variations in Size and Charge
Source: PLoS One. 2015 Sep 25;10(9):e0138892. doi: 10.1371/journal.pone.0138892 (PMC4583492; doi:10.1371/journal.pone.0138892)
Supplement: S2 Table — (DOCX) [file pone.0138892.s007.docx]

**S2 Table**. Unique proteins identified in each spot from 2DE of nickel isolated samples from two sea urchins

| **Spot numbers** | | **GenBank Accession number** | **Matches to the *S. purpuratus* protein database^1^** | **Sequential amino acids with ≤1 intervening amino acid^2^** | |
| --- | --- | --- | --- | --- | --- |
| **Animal 7** | **Animal 9** |  |  | **His** | **His, Asp, Gly Lys Ser** |
| **Immune Response** | | | |  |  |
| 6, 13 | 8, 9, 11 | 118421677 | Sp185/333 | 5 | 9 |
| 6 | 8, 9, 11 | 167046317 | Sp185/333 *E2* delta | 5 | 9 |
| 10 |  | 118421410 | Sp185/333, partial | 5 | 9 |
| 11 |  | 118421684 | Sp185/333 | 5 | 5 |
| 6, 9 | 8-10 | 75699917 | 185/333 | 5 | 9 |
| 4, 6-11, 13-15 | 1-5, 7-11, 13 | 47551023 | complement component C3 precursor | 2 | 7 |
| 2, 8, 9, 13 |  | 780071468 | complement C3, partial | 2 | 4 |
| 9, 10 |  | 780087817 | complement C3 | 1 | 4 |
| 1, 2, 8, 9, 13 |  | 780019653 | complement C5 | 2 | 4 |
| 6, 9, 10 |  | 780099734 | cobra venom factor | 1 | 4 |
| 6-9 | 3, 10, 11, 13, 14 | 780183095 | complement C2 | 1 | 5 |
|  | 10 | 780032958 | complement C2 (low quality protein) | 1 | 6 |
| 10, 11 | 11 | 47825406 | complement related-long precursor | 1 | 7 |
| 8-11, 13 | 2-5, 8-11, 13 | 780061230 | α-2-macroglobulin | 2 | 8 |
| 6, 8, 13 |  | 47550947 | scavenger receptor cysteine-rich protein precursor | 1 | 5 |
| 6, 10 |  | 47550953 | scavenger receptor cysteine-rich protein precursor | 1 | 5 |
| 6 | 11 | 780183890 | soluble scavenger receptor cysteine-rich domain-containing protein SSC5D | 1 | 3 |
|  | 9 | 47551157 | scavenger receptor cysteine-rich protein type 12 precursor | 3 | 7 |
|  | 11 | 47551161 | scavenger receptor cysteine-rich protein precursor | 2 | 6 |
| 9, 10 |  | 780184437 | deleted in malignant brain tumors 1 protein | 1 | 5 |
| 5 |  | 780180450 | deleted in malignant brain tumors 1 protein-like | 1 | 6 |
| 7, 9, 11, 14 | 7 | 780120082 | deleted in malignant brain tumors 1 protein-like  (low quality protein) | 1 | 5 |
| 2 |  | 780045777 | deleted in malignant brain tumors 1 protein | 1 | 6 |
| 9 | 10 | 779995768 | deleted in malignant brain tumors 1 protein-like | 1 | 6 |
| 6 | 4, 5, 11, 13 | 779991261 | deleted in malignant brain tumors 1 protein | 1 | 7 |
|  | 2, 13 | 390362331 | deleted in malignant brain tumors 1 protein | 1 | 6 |
|  | 11 | 390362405 | deleted in malignant brain tumors 1 protein, partial | 1 | 5 |
|  | 8 | 780118749 | sushi, von Willebrand factor type A, EGF and pentraxin domain-containing protein 1 isoform X4 | 2 | 6 |
|  | 11, 13 | 780171337 | sushi, von Willebrand factor type A, EGF and pentraxin domain-containing protein 1 | 2 | 7 |
| 7-9 | 2, 3, 7, 9-11 | 47550955 | DD104 protein | 1 | 10 |
| **Clotting** | | | |  |  |
| 7-10, 13 | 10 | 47551235 | amassin precursor | 1 | 5 |
| 7 |  | 118601058 | amassin 4 precursor | 1 | 6 |
| 8 |  | 780116389 | noelin | 1 | 5 |
| 14 |  | 780021356 | coagulation factor X, partial | 1 | 5 |
| 9 | 4, 10 | 780078609 | coagulation factor X, partial | 1 | 8 |
|  | 7, 11 | 780153488 | coagulation factor XIII B chain | 1 | 5 |
| 8, 13 | 8, 10, 11 | 72010678 | arylsulfatase | 2 | 5 |
| 8, 9, 13 | 8, 11 | 780054561 | arylsulfatase isoform X2 | 2 | 12 |
| **Ion binding** | | | |  |  |
| 6, 7, 9 | 2, 5, 7-12, 14 | 390352404 | melanotransferrin | 1 | 6 |
| 10-12 | 13, 14 | 47551123 | major yolk protein precursor | 3 | 5 |
| 10 |  | 780120784 | hephaestin-like protein | 2 | 5 |
|  | 2 | 780139722 | calumenin-B-like | 1 | 4 |
| **ER or Golgi localization** | | | |  |  |
| 14 | 5 | 47550939 | calreticulin precursor | 1 | 7 |
| 6 | 5 | 47551041 | ER calcistorin precursor | 1 | 7 |
| 3 | 3 | 780180833 | protein disulfide-isomerase A3 | 2 | 6 |
| 4 |  | 780134531 | selenium-binding protein 1-A | 1 | 6 |
| **MSC** | | | |  |  |
| 10 |  | 780021362 | low-density lipoprotein receptor-related protein 4 | 2 | 6 |
| 9-11 | 4 | 780029668 | low-density lipoprotein receptor-related protein 4 | 2 | 9 |
| 6 |  | 780050812 | SCO-spondin | 1 | 6 |
| 11 | 13 | 780092610 | mucin-2 (low quality protein) | 2 | 6 |
| 10 |  | 780092617 | mucin-5B-like | 3 | 5 |
| 11 | 13 | 390347058 | neural-cadherin | 1 | 7 |
| 14 | 5, 11 | 780004639 | steroidogenic acute regulatory protein, mitochondrial-like | 1 | 8 |
|  | 10 | 779994202 | serine protease 27-like | 1 | 6 |
| 1 |  | 779989142 | protein ADP-ribosylarginine hydrolase-like isoform X2 | 1 | 8 |
|  | 3 | 779992585 | rab GDP dissociation inhibitor alpha | 3 | 6 |
|  | 10 | 780045756 | alkaline phosphatase, tissue-nonspecific isozyme | 1 | 4 |
| 3 |  | 46909265 | ATP synthase beta subunit, partial | 1 | 5 |
| **Cytoskeletal** | | | |  |  |
| 1-4, 6-10, 11, 13-15 | 1-5, 7, 8, 10, 11, 13 | 226564 | cytoskeletal actin IIb | 2 | 7 |
| 1-3, 5, 7, 11, 13-15 | 2, 3, 5, 8, 10, 11, 13 | 115918029 | actin-15B | 2 | 7 |
| 2-4, 13, 15 | 2, 4, 5, 8, 10, 11, 13 | 390341092 | actin, cytoskeletal 3B | 2 | 7 |
|  | 2 | 161430 | actin 2 protein | 2 | 7 |
|  | 2, 13 | 72006507 | actin-5C | 2 | 7 |
| 2 |  | 115746623 | actin, cytoplasmic | 2 | 5 |
| 1-3, 13-15 | 1, 2, 5-8, 10, 11, 13 | 780178325 | muscle actin isoform X1 | 2 | 5 |
| 7 |  | 780066389 | gelsolin-like protein 1 | 1 | 8 |
| 3, 4 | 3, 4 | 115891439 | gelsolin-like protein 2 | 2 | 7 |
| 4, 6, 14, 15 | 5 | 737523 | fascin | 1 | 5 |
| 3 |  | 780135840 | plastin-2-like, partial | 1 | 6 |
| 7 | 2 | 390346228 | tropomyosin isoform X2 | 1 | 6 |
| 7 | 2, 3, 7 | 115725464 | short-chain collagen C4 | 1 | 5 |
| 4 |  | 72171507 | tubulin alpha-1 chain | 1 | 6 |
|  | 3, 4 | 780036755 | tubulin alpha-1 chain isoform X5 | 1 | 6 |
| 3-5 | 3-5 | 390349562 | tubulin beta chain isoform X1 | 1 | 6 |
| **Unknown** | | | |  |  |
| 6, 14 |  | 780164384 | uncharacterized protein LOC584567 isoform X2 | 2 | 6 |
|  | 2, 7 | 780086590 | uncharacterized protein LOC105442755 | 2 | 7 |
| 6, 10 | 3, 8 | 780075696 | uncharacterized protein LOC100894028 | 1 | 6 |
| 7 |  | 780029968 | uncharacterized protein LOC105439352 | 2 | 4 |
|  | 11 | 390367087 | uncharacterized protein LOC100892672 | 2 | 5 |
| 7 | 2, 3, 7 | 115673141 | uncharacterized protein LOC581886 | 1 | 5 |
| 7 |  | 115923317 | uncharacterized protein LOC577534 | 1 | 5 |
| 6, 7 | 7-10, 11 | 390367638 | uncharacterized protein LOC100893255 | 2 | 5 |
| 14 | 5, 11 | 780004674 | uncharacterized protein LOC585309 | 1 | 5 |
|  | 3 | 390356396 | uncharacterized protein LOC754270 isoform X2 | 1 | 13 |
|  | 13 | 780162231 | uncharacterized protein LOC753895 | 2 | 6 |

^1^Searches were conducted against the NCBI protein database for *Strongylocentrotus purpuratus* containing 38,417 entries, as of May 2015, using Proteome Discoverer Percolator software (24, 25). Note that the same protein is often represented redundantly in the database. For the complete data set generated from Percolator, see S3 Table.

^2^Fuzzpro (EMBOS) was used to search for amino acid patterns from FASTA protein sequences.
